# Probing the diabetes and colorectal cancer relationship using gene – environment interaction analyses

**DOI:** 10.1038/s41416-023-02312-z

**Published:** 2023-06-26

**Authors:** Niki Dimou, Andre E. Kim, Orlagh Flanagan, Neil Murphy, Virginia Diez-Obrero, Anna Shcherbina, Elom K. Aglago, Emmanouil Bouras, Peter T. Campbell, Graham Casey, Steven Gallinger, Stephen B. Gruber, Mark A. Jenkins, Yi Lin, Victor Moreno, Edward Ruiz-Narvaez, Mariana C. Stern, Yu Tian, Kostas K. Tsilidis, Volker Arndt, Elizabeth L. Barry, James W. Baurley, Sonja I. Berndt, Stéphane Bézieau, Stephanie A. Bien, D. Timothy Bishop, Hermann Brenner, Arif Budiarto, Robert Carreras-Torres, Tjeng Wawan Cenggoro, Andrew T. Chan, Jenny Chang-Claude, Stephen J. Chanock, Xuechen Chen, David V. Conti, Christopher H. Dampier, Matthew Devall, David A. Drew, Jane C. Figueiredo, Graham G. Giles, Andrea Gsur, Tabitha A. Harrison, Akihisa Hidaka, Michael Hoffmeister, Jeroen R. Huyghe, Kristina Jordahl, Eric Kawaguchi, Temitope O. Keku, Susanna C. Larsson, Loic Le Marchand, Juan Pablo Lewinger, Li Li, Bharuno Mahesworo, John Morrison, Polly A. Newcomb, Christina C. Newton, Mireia Obon-Santacana, Jennifer Ose, Rish K. Pai, Julie R. Palmer, Nikos Papadimitriou, Bens Pardamean, Anita R. Peoples, Paul D. P. Pharoah, Elizabeth A. Platz, John D. Potter, Gad Rennert, Peter C. Scacheri, Robert E. Schoen, Yu-Ru Su, Catherine M. Tangen, Stephen N. Thibodeau, Duncan C. Thomas, Cornelia M. Ulrich, Caroline Y. Um, Franzel J. B. van Duijnhoven, Kala Visvanathan, Pavel Vodicka, Ludmila Vodickova, Emily White, Alicja Wolk, Michael O. Woods, Conghui Qu, Anshul Kundaje, Li Hsu, W. James Gauderman, Marc J. Gunter, Ulrike Peters

**Affiliations:** 1 Nutrition and Metabolism Branch, International Agency for Research on Cancer, Lyon, France; 2 Division of Biostatistics, Department of Population and Public Health Sciences, Keck School of Medicine, University of Southern California, Los Angeles, CA USA; 3 Unit of Biomarkers and Susceptibility, Oncology Data Analytics Program, Catalan Institute of Oncology, Barcelona, 08908 Spain; 4 Colorectal Cancer Group, ONCOBELL Program, Bellvitge Biomedical Research Institute, Barcelona, 08908 Spain; 5 Consortium for Biomedical Research in Epidemiology and Public Health, Barcelona, 08908 Spain; 6 Department of Clinical Sciences, Faculty of Medicine, University of Barcelona, Barcelona, 08908 Spain; 7 Department of Genetics, Stanford University, Stanford, CA USA; 8 Department of Computer Science, Stanford University, Stanford, CA USA; 9 School of Public Health, Imperial College London, London, United Kingdom; 10 Department of Hygiene and Epidemiology, University of Ioannina School of Medicine, Ioannina, Greece; 11 Department of Epidemiology and Population Health, Albert Einstein College of Medicine, Bronx, NY USA; 12 Department of Public Health Sciences, Center for Public Health Genomics, Charlottesville, VA USA; 13 Lunenfeld Tanenbaum Research Institute, Mount Sinai Hospital, University of Toronto, Toronto, ON Canada; 14 Center for Precision Medicine, Department of Medical Oncology and Therapeutics Research, City of Hope National Medical Center, Duarte, CA USA; 15 Centre for Epidemiology and Biostatistics, Melbourne School of Population and Global Health, The University of Melbourne, Melbourne, Victoria Australia; 16 Public Health Sciences Division, Fred Hutchinson Cancer Center, Seattle, WA USA; 17 Oncology Data Analytics Program, Catalan Institute of Oncology-IDIBELL, L’Hospitalet de Llobregat, Barcelona, Spain; 18 CIBER Epidemiología y Salud Pública (CIBERESP), Madrid, Spain; 19 ONCOBEL Program, Bellvitge Biomedical Research Institute (IDIBELL), L’Hospitalet de Llobregat, Barcelona, Spain; 20 Department of Nutritional Sciences, University of Michigan School of Public Health, Ann Arbor, MI USA; 21 Department of Population and Public Health Sciences & USC Norris Comprehensive Cancer Center, Keck School of Medicine, University of Southern California, Los Angeles, CA USA; 22 Division of Cancer Epidemiology, German Cancer Research Center (DKFZ), Heidelberg, Germany; 23 School of Public Health, Capital Medical University, Beijing, China; 24 Division of Clinical Epidemiology and Aging Research, German Cancer Research Center (DKFZ), Heidelberg, Germany; 25 Department of Epidemiology, Geisel School of Medicine at Dartmouth, Hanover, NH USA; 26 Bioinformatics and Data Science Research Center, Bina Nusantara University, Jakarta, Indonesia; 27 BioRealm LLC, Walnut, CA USA; 28 Division of Cancer Epidemiology and Genetics, National Cancer Institute, National Institutes of Health, Bethesda, MD USA; 29 Nantes Université, CHU Nantes, Service de Génétique médicale, F-44000 Nantes, France; 30 Leeds Institute of Cancer and Pathology, University of Leeds, Leeds, UK; 31 Division of Preventive Oncology, German Cancer Research Center (DKFZ) and National Center for Tumor Diseases (NCT), Heidelberg, Germany; 32 German Cancer Consortium (DKTK), German Cancer Research Center (DKFZ), Heidelberg, Germany; 33 Computer Science Department, School of Computer Science, Bina Nusantara University, Jakarta, Indonesia; 34 Colorectal Cancer Group, ONCOBELL Program, Bellvitge Biomedical Research Institute (IDIBELL), L’Hospitalet de Llobregat, 8908 Barcelona, Spain; 35 Division of Gastroenterology, Massachusetts General Hospital and Harvard Medical School, Boston, MA USA; 36 Channing Division of Network Medicine, Brigham and Women’s Hospital and Harvard Medical School, Boston, MA USA; 37 Clinical and Translational Epidemiology Unit, Massachusetts General Hospital and Harvard Medical School, Boston, MA USA; 38 Broad Institute of Harvard and MIT, Cambridge, MA USA; 39 Department of Epidemiology, Harvard T.H. Chan School of Public Health, Harvard University, Boston, MA USA; 40 Department of Immunology and Infectious Diseases, Harvard T.H. Chan School of Public Health, Harvard University, Boston, MA USA; 41 University Medical Centre Hamburg-Eppendorf, University Cancer Centre Hamburg (UCCH), Hamburg, Germany; 42 Medical Faculty Heidelberg, Heidelberg University, Heidelberg, Germany; 43 Department of General Surgery, University of Virginia School of Medicine, Charlottesville, VA USA; 44 Department of Family Medicine, University of Virginia, Charlottesville, VA USA; 45 Clinical & Translational Epidemiology Unit, Massachusetts General Hospital and Harvard Medical School, Boston, MA USA; 46 Department of Medicine, Samuel Oschin Comprehensive Cancer Institute, Cedars-Sinai Medical Center, Los Angeles, CA USA; 47 Cancer Epidemiology Division, Cancer Council Victoria, Melbourne, VIC Australia; 48 Precision Medicine, School of Clinical Sciences at Monash Health, Monash University, Clayton, VIC Australia; 49 Center for Cancer Research, Medical University of Vienna, Vienna, Austria; 50 Center for Gastrointestinal Biology and Disease, University of North Carolina, Chapel Hill, NC USA; 51 Institute of Environmental Medicine, Karolinska Institutet, Stockholm, Sweden; 52 University of Hawaii Cancer Center, Honolulu, HI USA; 53 School of Public Health, University of Washington, Seattle, WA USA; 54 Department of Population Science, American Cancer Society, Atlanta, GA USA; 55 Unit of Nutrition, Environment and Cancer, Cancer Epidemiology Research Program, Catalan Institute of Oncology (ICO-IDIBELL), Avda Gran Via Barcelona 199-203, 08908L’Hospitalet de Llobregat, Barcelona, Spain; 56 Huntsman Cancer Institute, University of Utah, Salt Lake City, UT USA; 57 Department of Population Health Sciences, University of Utah, Salt Lake City, UH USA; 58 Department of Laboratory Medicine and Pathology, Mayo Clinic Arizona, Scottsdale, AZ USA; 59 Slone Epidemiology Center at Boston University, Boston, MA USA; 60 Department of Public Health and Primary Care, University of Cambridge, Cambridge, UK; 61 Department of Epidemiology, Johns Hopkins Bloomberg School of Public Health, Baltimore, MD USA; 62 Department of Epidemiology, School of Public Health, University of Washington, Seattle, WA USA; 63 Research Centre for Hauora and Health, Massey University, Wellington, New Zealand; 64 Department of Community Medicine and Epidemiology, Lady Davis Carmel Medical Center, Haifa, Israel; 65 Ruth and Bruce Rappaport Faculty of Medicine, Technion-Israel Institute of Technology, Haifa, Israel; 66 Clalit National Cancer Control Center, Haifa, Israel; 67 Department of Genetics and Genome Sciences, Case Western Reserve University, Cleveland, OH USA; 68 Department of Medicine and Epidemiology, University of Pittsburgh Medical Center, Pittsburgh, PA USA; 69 Biostatistics Division, Kaiser Permanente Washington Health Research Institute, Seattle, WA USA; 70 SWOG Statistical Center, Fred Hutchinson Cancer Center, Seattle, WA USA; 71 Division of Laboratory Genetics, Department of Laboratory Medicine and Pathology, Mayo Clinic, Rochester, MN USA; 72 Division of Human Nutrition and Health, Wageningen University & Research, Wageningen, The Netherlands; 73 Department of Molecular Biology of Cancer, Institute of Experimental Medicine of the Czech Academy of Sciences, Prague, Czech Republic; 74 Institute of Biology and Medical Genetics, First Faculty of Medicine, Charles University, Prague, Czech Republic; 75 Faculty of Medicine and Biomedical Center in Pilsen, Charles University, Pilsen, Czech Republic; 76 Memorial University of Newfoundland, Discipline of Genetics, St. John’s, NL Canada; 77 Department of Biostatistics, University of Washington, Seattle, WA USA

**Keywords:** Colon cancer, Cancer genetics

## Abstract

**Background:**

Diabetes is an established risk factor for colorectal cancer. However, the mechanisms underlying this relationship still require investigation and it is not known if the association is modified by genetic variants. To address these questions, we undertook a genome-wide gene-environment interaction analysis.

**Methods:**

We used data from 3 genetic consortia (CCFR, CORECT, GECCO; 31,318 colorectal cancer cases/41,499 controls) and undertook genome-wide gene-environment interaction analyses with colorectal cancer risk, including interaction tests of genetics(G)xdiabetes (1-degree of freedom; d.f.) and joint testing of Gxdiabetes, G-colorectal cancer association (2-d.f. joint test) and G-diabetes correlation (3-d.f. joint test).

**Results:**

Based on the joint tests, we found that the association of diabetes with colorectal cancer risk is modified by loci on chromosomes 8q24.11 (rs3802177, *SLC30A8 –* OR_*AA*_: 1.62, 95% CI: 1.34–1.96; OR_*AG*_: 1.41, 95% CI: 1.30–1.54; OR_*GG*_: 1.22, 95% CI: 1.13–1.31; *p*-value_3-d.f._: 5.46 × 10^−11^) and 13q14.13 (rs9526201, *LRCH1 –* OR_*GG*_: 2.11, 95% CI: 1.56–2.83; OR_*GA*_: 1.52, 95% CI: 1.38–1.68; OR_*AA*_: 1.13, 95% CI: 1.06–1.21; *p*-value_2-d.f._: 7.84 × 10^−09^).

**Discussion:**

These results suggest that variation in genes related to insulin signaling (*SLC30A8*) and immune function (*LRCH1*) may modify the association of diabetes with colorectal cancer risk and provide novel insights into the biology underlying the diabetes and colorectal cancer relationship.

## Introduction

Colorectal cancer is the third most common cancer globally with an estimated number of 1.9 million new cases in 2020 [1]. The etiology of colorectal cancer involves a complex interplay between genetic and environmental determinants. Currently, around 140 genetic variants have been identified by genome-wide association studies (GWAS) explaining ~12% of the variability in colorectal cancer risk [2, 3]. However, limited research has been conducted to understand the interaction between genetic and environmental/lifestyle risk factors on the risk of colorectal cancer. Understanding how genetic variation may modify the association of environmental and lifestyle exposures with colorectal cancer risk may potentially uncover novel biological pathways underlying disease etiology and contribute to the development of prevention strategies.

Type 2 diabetes (T2D), the most common form of diabetes, is an established risk factor for colorectal cancer [4]. The biological mechanisms that underlie the association between T2D and colorectal cancer risk are not fully understood but likely entail exposure to hyperinsulinemia and insulin resistance as well as hyperglycemia, which often precede onset of T2D [5]. However, it is possible that other, yet-to-be recognized, molecular pathways mediate the T2D-colorectal cancer relationship.

Gene-environment interaction (GxE) studies have been employed to investigate whether genetic variants modify the association of diet, lifestyle, and drugs with colorectal cancer [6]. A previous GxE analysis of diabetes and risk of colorectal cancer was limited by small sample size and was focused on candidate genes [7]. To provide further insights into the molecular pathways of diabetes with colorectal cancer risk, we undertook a large-scale genome-wide GxE analysis that tested for interactions between common and rare variants and diabetes in 31,318 colorectal cancer cases and 41,499 controls.

## Methods

### Study participants

For this gene-environment interaction analysis, we used data from 48 studies described elsewhere [2, 3, 8, 9] (Supplementary Table 1). Briefly, we combined genetic and epidemiologic data from studies participating in the Colon Cancer Family Registry (CCFR), the Genetics and Epidemiology of Colorectal Cancer Consortium (GECCO), and the Colorectal Cancer Transdisciplinary Study (CORECT) with individuals of European ancestry. For cohort studies and clinical trials, nested case-control sets were assembled. Controls were matched on factors such as age, sex, race, and enrollment date or trial group (only in SELECT and a subset of WHI study), when applicable. Colorectal adenocarcinoma cases were confirmed by medical records, pathology reports, or death-certificate information. All studies were approved by the relevant research ethics committee or institutional review board.

Analyses were limited to individuals of European ancestry, based on self-reported race and clustering of principal components with 1000 Genomes EUR superpopulation. We further excluded individuals based on cryptic relatedness or duplicates (prioritizing cases and/or individuals genotyped on the better platform) (*N* = 2284), and genotyping/imputation errors (*N* = 9). When two cases were from the same matching pair, we kept the younger case (*N* = 71). Additionally, individuals were excluded if they had missing diabetes status (*N* = 2958), with age, gender and colorectal case/control status being largely unrelated to diabetes missingness. The final pooled sample size was 31,318 colorectal cancer cases and 41,499 controls.

### Harmonization of epidemiologic data

Information on demographics and potential risk factors were collected by self-report using in-person interviews and/or structured questionnaires [10]. Individuals with diabetes were defined using a binary self-reported diagnosis of the disease (not explicitly defined if diabetes is Type I or Type II). Given that Type I diabetes is rare, it is most likely that the majority of the participants live with Type II diabetes (although, any misclassification cannot be ruled out). Data were collected and centralized at the GECCO coordinating center (Fred Hutchinson Cancer Center). Briefly, data harmonization consisted of a multi-step procedure, where common data elements (CDEs) were defined a priori. Study questionnaires and data dictionaries were examined and, through an iterative process of communication with data contributors, elements were mapped to these CDEs. Definitions, permissible values, and standardized coding were implemented into a single database via SAS and T-SQL. The resulting data were checked for errors and outlying values within and between studies.

### Genotyping, quality assurance/quality control and imputation

Detailed information on genotyping, imputation, and quality control are presented elsewhere [2, 3]. In brief, genotyped variants were excluded based on deviation from Hardy–Weinberg Equilibrium (*p*-value < 1 × 10^−4^), low call rate (<95–98%), discrepancies between reported and genotypic sex, discordant calls between duplicates. Autosomal variants of all studies were imputed to the Haplotype Reference Consortium (HRC) r1.1 (2016) panel using the University of Michigan Imputation Server [11] and converted into a binary format for data management and analyses using R package BinaryDosage [12]. Imputed variants were excluded if they had low imputation quality (*R*^2^ < 0.8). After quality control, a total of over 7.2 million variants were used for the gene-environment interaction analysis for common variants and 25,216 gene sets for rare variants (i.e. with minor allele frequency below 1%).

### Statistical methods

#### Association of diabetes with colorectal cancer risk

To evaluate the main association of diabetes with colorectal cancer risk, each study was analyzed separately using logistic regression models. Study-specific results were combined using a random-effects meta-analysis (Hartung–Knapp) to obtain summary odds ratios (ORs) and 95% confidence intervals (CIs) across studies [13]. We calculated the heterogeneity *p*-values using Cochran’s Q statistic [14] and funnel plots were used to identify studies with outlying ORs for potential exclusion and sensitivity analyses.

#### GxE analyses for common variants

We performed genome-wide interaction scans using GxEScanR [15]. Our primary inferences are based on the standard 1-degrees of freedom (d.f.) GxE test, the 2-step EDGE approach [16], and the 3-d.f. joint test (joint association of main genetic effect on colorectal cancer, G-E association, and GxE interaction) [17]. Compared to the 1-d.f., the 3-d.f. joint test has higher power to detect GxE interactions when they exist, while accommodating gene-disease and gene-exposure associations [17]. The two-step method reduces the burden of multiple testing by preserving the statistical power, mainly through the initial filtering step [16]. We applied a family-wise error rate for each set to 0.05/3 to control for multiple testing. We note that this approach is conservative as these testing approaches are somewhat correlated.

We implemented a hybrid two-step method that prioritizes potential interaction loci by weighting GxE tests (step 2) based on the ranks of an independent test statistic (step 1). Step 1 tests include a joint test referred to as the EDGE statistic [16] of the marginal association of each variant with risk of colorectal cancer [18] and the association between each variant with diabetes in the combined case-control sample [19]. Our approach modifies the original weighted hypothesis testing framework [20] by accounting for linkage disequilibrium in controlling for type I error [21] (details are provided in the Supplementary Methods).

In secondary analyses, we used the 2-d.f. test that evaluates simultaneously the main genetic effect and the GxE interaction and has been shown to improve power to detect susceptibility loci under a wide range of circumstances by accounting for GxE interactions [22, 23]. A *p*-value < 5 × 10^−8^ was used to declare statistical significance, with the qualification that these findings were secondary. All tests were two-sided.

Imputed variant dosages were modeled as continuous variables [24]. All analyses were adjusted for age at baseline, sex, study/genotyping platform, and the first three principal components to account for potential population structure. Statistically significant interactions were further adjusted for body mass index (BMI) because it is a potential confounder in the diabetes-colorectal cancer association [25]. A pooled analysis is preferred over a meta-analytical approach as the latter is prone to violation of normality assumptions when effect estimates of studies with small sample sizes are combined.

For statistically significant findings, we estimated stratified ORs by modeling the association between diabetes and colorectal cancer risk stratified by genotype and association of the per-allele increase in genotype and colorectal cancer risk stratified by diabetes status. We assessed the extent of genomic inflation by quantile-quantile (Q-Q) plots and by calculating the genomic inflation factor (lambda). As lambda scales according to sample size, we also calculated lambda_1000_, which scales the genomic inflation factor to an equivalent study of 1000 cases and 1000 controls [26, 27].

To present 2-d.f., 3-d.f. test, and two-step-method results, we created additional plots after removing known GWAS colorectal cancer loci (and variants in close proximity ±2MB with correlation *r*^2^ > 0.2) [2] to ensure the overall significance is not driven merely by the main genetic effect on colorectal cancer.

Regional plots for all statistically significant findings were generated using LocusZoom v1.3 [28]. Measures of linkage disequilibrium (LD) were estimated using our controls. Possible eQTL relationships were explored using the Genotype-Tissue Expression (GTEx V8) and the University of Barcelona and University of Virginia genotyping and RNA sequencing project (BarcUVa-Seq) datasets [29]. The BarcUVa-Seq data has data on diabetes status of 410 participants which we used to test interactions between the genetic variants and diabetes on gene expression.

#### Prediction of regulatory impact of candidate non-coding variants

We used ATAC-seq, DNASE-seq, H3K27ac histone ChIP-seq, and H3K4me1 histone ChIP-seq datasets of primary tissue from healthy colon and tumor primary tissue samples from Scacheri et al. [30], as well as from three colorectal cancer cell lines (SW480, HCT116, COLO205). These datasets were processed through ENCODE ATAC-seq/DNASE-seq [31] and histone ChIP-seq pipelines [32] to perform alignment and peak calling. Dataset sources are indicated in Supplementary Table 2. −log10(*p*-value) tracks were extracted from the MACS2 step of the pipeline for visualization in genome browsers. Irreproducible Discovery Rate (IDR) [33] peak calls for ATAC-seq and DNASE-seq datasets, as well as naive overlap peak calls for histone ChIP-seq datasets, were determined from the ENCODE pipelines. The pyGenomeTracks [34] software package was used to visualize chromatin accessibility across the functional datasets and to plot −log10(*p*-value) signal tracks. Peaks across samples from the same assay were concatenated across datasets, cropped to within 200 bp centered on the peak summit, and merged using bedtools [35] merge.

Gapped k-mer support vector machine models (LS-GKM) (v0.1.0) with a center-weighted GKM kernel were trained to classify chromatin accessible regions against genomic background regions as a function of their underlying DNA sequences [36]. Default parameters were utilized. Support vector machines (SVMs) were trained via 10-fold cross-validation, where groups of chromosomes were split into folds (Supplementary Table 3). Separate SVM models were trained on DNase-seq data from Supplementary Table 2 with samples pooled across assays as described above [30] (details are provided in the Supplementary Methods).

#### GxE analyses for rare variants

As power for rare variant testing – and particularly GxE testing – tends to be low, we conducted GxE testing only for rare variants as a secondary analysis. We performed interaction tests of diabetes and aggregated rare variant sets at the gene and enhancer level (details are provided in the Supplementary Methods) using the Mixed effects Score Tests for interactions (MiSTi) method [37]. This unified hierarchical regression framework combines the burdenxE (all variants with a MAF of <1% were included in the variant sets) as fixed effect and heterogeneous GxE effects as random effects. We considered a Fisher’s combination approach under MiSTi (fMiSTi) to discover GxE interactions [37], adjusting for age at baseline, sex, study, genotyping platform, and the first three principal components. Since 25,000 genes were tested and this was a secondary analysis, interactions with *p*-value < 2 × 10^−6^ (*a* = 0.05/25,000) were considered suggestively significant. The MiSTi R package was used for rare variants interaction analyses [37].

## Results

Overall, diabetes was associated with a significantly higher risk of colorectal cancer (OR: 1.36, 95% CI: 1.23–1.51, Table 1), with similar results found in cohort and case-control studies. This association showed statistically significant between-study heterogeneity (Cochran’s Q *p*-value: <0.001; *I*^2^ = 48%, Supplementary Fig. S1). However, there were no strong outlying studies (Supplementary Fig. S2).

**Table 1:** Characteristics of the study participants included in the gene-diabetes interaction analysis for colorectal cancer risk.

	Colorectal cancer cases (*N* = 31,318)	Controls (*N* = 41,499)
Age (years)^a^	63.3 (±10.1)	62.1 (±8.97)
Sex		
Women	47.4%	49.4%
Tumor site		
Proximal	29.2%	
Distal	24.9%	
Rectal	26.0%	
Missing	19.9%	
Body mass index (kg/m^2^)^a^	27.4 (±4.86)	27.0 (±4.61)
Missing	6.6%	3.5%
Height (cm)^a^	170 (±9.61)	169 (±9.59)
Missing	1.7%	0.7%
Family history of colorectal cancer		
Yes	13.5%	10.5%
Missing	18.6%	26.9%
Education (highest completed)		
Less than High School	24.8%	20.1%
College/Graduate School	26.5%	31.7%
Missing	9.3%	10.0%
Smoking status		
Ever	53.2%	49.9%
Missing	4.5%	1.8%
Post-menopausal hormone use^b^		
Yes	25.5%	29.6%
Missing	25.9%	19.9%
Regular aspirin or NSAID use		
Yes	25.2%	32.1%
Missing	25.1%	16.7%
Diabetes		
Yes	11.4%	7.7%
Red meat consumption		
Highest quartile	18.7%	16.0%
Missing	15.4%	8.5%
Processed meat consumption		
Highest quartile	13.8%	11.8%
Missing	25.5%	16.4%

*NSAID* non-steroidal anti-inflammatory drugs.

^a^Mean and standard deviation.

^b^Among women only.

In our primary analysis we found that the association between diabetes and colorectal cancer risk was modified by variants on chromosome 8q24.11 within the *SLC30A8* gene based on the 3-d.f. joint test, with rs3802177 being the genetic variant showing the most significant effect (*p*-value: 5.46 × 10^−11^, Supplementary Figs. S3A, S4A, Table 2). This result was robust in a sensitivity analysis accounting for BMI (Table 2). Although this variant was not directly associated with colorectal cancer (*P*-value: >0.05), we observed a strong association with diabetes (*P*-value: 4.90 × 10^−10^), and an interaction with diabetes for colorectal cancer risk (*P*-value: 7.49 × 10^−04^). When we stratified by genotype of rs3802177 (with *A* as variant allele), we observed that the OR for diabetes vs. colorectal cancer among those carrying the *AA* genotype was the largest: 1.62, 95% CI: 1.34–1.96, *P*-value: 7.5 × 10^−07^, compared with OR: 1.41; 95% CI: 1.30–1.54; *P*-value: 6.2 × 10^−16^ among those carrying the *AG* genotype and, OR: 1.22; 95% CI: 1.13–1.31; *P*-value: 2.4 × 10^−07^ for those carrying the *GG* genotype. When stratifying by diabetes status, the risk of developing colorectal cancer per *G* allele was not statistically significant in those without diabetes (OR: 1.00; 95% CI: 0.98–1.03; *P*-value: 8.2 × 10^−01^) but was inverse among those with diabetes (OR: 0.87; 95% CI: 0.80–0.94; *P*-value: 5.4 × 10^−04^) (Table 3). The full GxE results are available in Table 3. We did not identify any statistically significant interactions using the traditional logistic regression or the 2-step approach. Genomic inflation for 1-d.f. GxE was minimal (lambda = 1.008; lambda_1000_ = 1.000).

**Table 2:** Statistically significant results of the gene-environment interaction analyses for diabetes and single genetic variants for colorectal cancer risk.

Variant	Chr	BP position	Closest gene	Annotation	Reference allele	Alternate allele	Alternate allele frequency	Method	*P* (D|G)^a^	*P* (E|G)^b^	*P* (GxE)^c^	*P*-value^d^ (Covariate set 1)^e^	*P*-value^d^ (Covariate set 2)^f^
Primary GxE testing													
rs3802177	8	118185025	*SLC30A8*	3’ UTR	*G*	*A*	0.31	3-d.f. joint test	4.05 × 10^−01^	4.90 × 10^−10^	7.49 × 10^−04^	5.46 × 10^−11^	1.01 × 10^−10^
Secondary GxE testing													
rs9526201	13	47191972	*LRCH1*	intron	*G*	*A*	0.82	2-d.f. joint test	1.87 × 10^−04^	NA	1.33 × 10^−06^	7.84 × 10^−09^	8.82 × 10^−09^

*Chr* chromosome, *BP Position* base pair position based on NCBI Build 37, *d.f.* degrees of freedom, *UTR* Untranslated region, *NA* not applicable.

^a^*P*-value corresponds to the association between genetic variants and colorectal cancer.

^b^*P*-value corresponds to the association between genetic variants and diabetes.

^c^*P*-value corresponds to the interaction between genetic variants and diabetes on risk of colorectal cancer.

^d^*P*-values correspond to each method used to test for interactions between genetic variants and diabetes.

^e^Covariate set 1: age at baseline, sex, study, genotyping platform, and the first three principal components (Colorectal cancer cases = 31,318, Controls = 41,499).

^f^Covariate set 2: covariate set 1 plus additional adjustment for body mass index.

**Table 3:** Stratified analysis for gene-diabetes interactions for colorectal cancer that were statistically significant.

Primary GxE testing: rs3802177, chromosome 8, *SLC30A8*											
	*AA*			*AG*			*GG*			*per G allele stratified by diabetes*	
	#cases /#controls	OR (95% CI)	*p*-value	#cases /#controls	OR (95% CI)	*p*-value	#cases /#controls	OR (95% CI)	*p*-value	OR (95% CI)	*p*-value
Diabetes = No	2605/3640	1.00 (Ref.)	–	11848/16321	1.00 (0.95–1.06)	9.3 × 10^−01^	13307/18333	1.01 (0.95–1.07)	8.5 × 10^−01^	1.00 (0.98–1.03)	8.2 × 10^−01^
Diabetes = Yes	300/227	1.62 (1.34–1.96)	7.5 × 10^−07^	1496/1285	1.42 (1.29–1.56)	1.2 × 10^−12^	1762/1694	1.23 (1.12–1.34)	7.5 × 10^−06^	0.87 (0.80–0.94)	5.4 × 10^−04^
OR for diabetes stratified by genotype	–	1.62 (1.34–1.96)	7.5 × 10^−07^	–	1.41 (1.3–1.54)	6.2 × 10^−16^	–	1.22 (1.13–1.31)	2.4 × 10^−07^		
**Secondary GxE testing: rs9526201, chromosome 13,** ***LRCH1***											
	***GG***			***GA***			***AA***			***per A allele stratified by diabetes***	
	#cases /#controls	OR (95% CI)	*p*-value	#cases /#controls	OR (95% CI)	*p*-value	#cases /#controls	OR (95% CI)	*p*-value	OR (95% CI)	*p*-value
Diabetes = No	859/1342	1.00 (Ref.)	–	7958/11639	1.06 (0.96–1.17)	2.2 × 10^−01^	18943/25313	1.15 (1.05–1.27)	2.9 × 10^−03^	1.08 (1.05–1.11)	4.7 × 10^−7^
Diabetes = Yes	133/92	2.11 (1.56–2.83)	8.9 × 10^−07^	1068/902	1.62 (1.42–1.84)	7.5 × 10^−13^	2358/2212	1.3 (1.16–1.46)	1.8 × 10^−09^	0.85 (0.77–0.93)	5.9 × 10^−4^
OR for diabetes stratified by genotype	–	2.11 (1.56–2.83)	8.9 × 10^−07^	–	1.52 (1.38–1.68)	1.1 × 10^−16^	–	1.13 (1.06–1.21)	3.8 × 10^−09^		

Number of case/control counts were calculated by imputed genotype probabilities. Analyses were adjusted for age at baseline, sex, study, genotyping platform, and the first three principal components.

Odds ratios (OR) and 95% confidence interval (CI) are shown for association of diabetes with colorectal cancer risk stratified by genotype (rs3802177 and rs9526201) and for association of genotype with colorectal cancer risk stratified by diabetes.

In our secondary 2-d.f. joint test, we identified that the association between diabetes and colorectal cancer risk is modified by a locus on chromosome 13q14.13 within the *LRCH1* gene, with genetic variant rs9526201 showing the most significant effect (*p*-value: 7.84 × 10^−09^, Supplementary Figs. S3B and S4B, Table 2). This result was robust in a sensitivity analysis accounting for BMI (Table 2). As can be seen in Table 2, the *p*-value for the genetic variant-diabetes interaction was 1.33 × 10^−06^ and the association between genetic variants and colorectal cancer was 1.87 × 10^−04^, resulting in a combined significant 2-d.f. test statistic. When we stratified the association between diabetes and colorectal cancer by genotype of rs9526201 (with *G* as variant allele), we observed a substantially stronger association among those carrying the *GG* genotype with an OR of 2.11 (95% CI: 1.56–2.83, *P*-value: 8.9 × 10^−07^), compared with an OR of 1.52 (95% CI: 1.38–1.68; *P*-value: 1.1 × 10^−16^) among those carrying the *GA* genotype and, an OR of 1.13 (95% CI: 1.06–1.21; *P*-value: 3.8 × 10^−09^) among those carrying the *AA* genotype. When stratifying by diabetes status, the risk of developing colorectal cancer increased per *A* allele in those without diabetes (OR: 1.08; 95% CI: 1.05–1.11; *P*-value: 4.7 × 10^−7^) but decreased in those with diabetes (OR: 0.85; 95% CI: 0.77–0.93; *P*-value: 5.9 × 10^−4^) (Table 3).

We did not identify any statistically significant GxE interactions when testing gene sets with rare variants.

We used two independent sources of eQTLs to evaluate the regulatory role of rs3802177 and rs9526201 variants on gene expression. Variant rs3802177 was not associated with gene expression in GTEx data; however, there was a suggestive eQTL in BarcUVa-Seq data that regulates expression of *AARD*, with the *G* allele associated with increased expression (*β*: 0.14, *P*-value: 4.7 × 10^−2^) (Supplementary Table S4). Also, variants in LD *R*^2^ > 0.5 with rs3802177 were suggestive eQTLs in GTEx transverse colon data that are associated with the expression of *AARD* (Supplementary Table S5). For the BarcUVa data, we assessed the diabetes status of participants who provided this information (N: 49 individuals with diabetes; N: 361 without diabetes) and tested for interactions between the variant and diabetes on gene expression. There was no evidence of a statistically significant interaction (*P*-values > 0.05) of variant rs3802177 (or variants in LD *R*^2^ > 0.5 with rs3802177) with diabetes in relation to *SLC30A8* gene expression in BarcUVa-Seq data (or any gene within 1 Mb of rs3802177).

Variant rs9526201 is an eQTL in the GTEx V8 compendium that influences the expression of *LRCH1* in 8 non-colorectal tissues (Supplementary Table S6) and variants correlated with rs9526201 are suggestive eQTLs for *LRCH1* based on GTEx transverse colon tissue (Supplementary Table S5). Also, variant rs9526201 is a suggestive eQTL in normal colon tissue (from the BarcUVa-Seq data) that regulates expression of *RUBCNL*, with the *A* allele associated with increased expression (*β*: 0.17, *p*-value: 1.3 × 10^−2^) (Supplementary Table S4). We found a suggestive interaction (*P*-value: 0.02) of variant rs9534444 (LD *R*^2^: 0.52 with rs9526201) with diabetes in relation to *LRCH1* gene expression (Supplementary Fig. S5).

Functional annotation analyses showed no evidence of enhancer activity for the variant rs3802177 or variants correlated with this variant (Supplementary Fig. S6A). However, the variant rs9526201 in the *LRCH1* gene is associated with pronounced enhancer activity in colon tumor and cancer cell lines (Supplementary Fig. S6B) and is in proximity with several variants that are located in open chromatin, suggesting enhancer activity in normal colon tissues, colorectal cancer cell lines, and several tissues (Supplementary Table S7).

We expanded our candidate set of variants to include variants in LD, in a 500 kb window around rs3802177 and rs9526201 variants (LD *R*^2^ > 0.20) and used gkmSVM models to predict variant allelic effects on chromatin accessibility (Supplementary Fig. S7). For rs3802177 and rs9526201, the models showed a weak difference in predicted chromatin accessibility between the reference and alternate alleles (Supplementary Table S8). We found a borderline difference in predicted chromatin accessibility between the alternate G allele and the reference C (ISM score = −1.148 in HCT116) for variant rs9534444 (LD *R*^2^: 0.52 with rs9526201; Supplementary Table S8).

GkmExplain analysis of rs3802177 and rs9526201 showed that there a was weak allelic effect in healthy tissue, tumor tissue, and cancer cell lines (Supplementary Fig. S8). G to A variation in rs3802177 disrupts IRX3, leading to an increased probability of chromatin accessibility with the A allele whereas A to G allelic variation in rs9526201 completes a RUNX1 motif, leading to decreased probability of chromatin accessibility with the G allele (Supplementary Fig. S8). For variant rs9534444 which is the highest-effect variant, in LD with rs9526201, results suggested that C to G variation disrupts motifs ZN341, MYF5, and PRGR.

## Discussion

In this large genome-wide GxE interaction analysis involving more than 30,000 colorectal cancer cases, we found that the association of diabetes status with colorectal cancer was modified by common genetic variants located within the *SLC30A8* and *LRCH1* genes. The mechanisms linking diabetes with colorectal cancer are not fully understood. Dysregulation of insulin and glucose metabolism are important candidate mechanisms and hyperinsulinemia itself has been causally linked to colorectal cancer development [38, 39]; however, the precise mechanisms linking these phenomena are not clear. The findings of this analysis may provide biological insights into the established link between diabetes and colorectal cancer.

We found that the association of diabetes with colorectal cancer risk was modified by variants located in the *SLC30A8* gene. These genetic variants were not statistically significantly associated with gene expression in GTEx and only a weak eQTL has been observed in colorectal tissue for the *AARD* gene. Furthermore, the genetic variants were not located within predicted enhancer regions and we observed only weak evidence for allele-specific effects. Given the limited functional evidence, we focused on the closest gene, *SLC30A8*, which encodes a zinc transporter, ZnT8, that regulates zinc accumulation in the beta cells of the pancreas [40]. Zinc is implicated in the phosphorylation of the insulin receptor beta-subunit and phosphatidylinositol 3-kinase (PI3K)/serine/threonine-specific protein kinase (Akt) signaling pathway [41, 42]. Dysregulation of the PI3K/AKT pathway is associated with diabetes development [43] and with anti-apoptotic effects in colorectal cancer cells [44, 45]. Our top hit, rs3802177 in the *SLC30A8* gene is in LD (*R*^2^ = 1) with rs13266634, which was associated with diabetes risk in a previous GWAS [46] (as well as in our analysis) and has also been shown to modify insulin secretion [47]. In summary, although we did not find strong functional genomic support for this highly significant association, the genetic variant is located within *SLC30A8* which is a strong candidate gene for modifying the diabetes-colorectal cancer association.

We also observed that the association of diabetes with colorectal cancer risk was modified by genetic variants located in *LRCH1*. eQTL as well as gene-expression analysis for the variantxdiabetes interaction suggests that *LRCH1* might represent the target gene regulating expression and transcription. The variants in LD with rs9526201 are located in enhancer peaks and we observed borderline significant allele-specific effects for variant rs9534444. For rs9534444, a C-to-G mutation disrupts the motif “TGGAAGAGCAGATGG”, which the TomTom software presents as a significant match to the know binding motifs of the ZN341, MYF5, PRGR transcription factors. The loss of function in response to the C-to-G mutation was observed in all 5 datasets profiled via SVM, with the strongest effects observed in the HCT116 cell line. LRCH1 is known to interact with DOCK8 to restrain the guanine-nucleotide exchange factor activity of DOCK8, resulting in the inhibition of Cdc42 activation and T cell migration [48]. Cdc42 activation has been related to several malignancies, including colorectal cancer [49]. Increased Cdc42 levels have been associated with colorectal cancer progression by promoting colorectal cancer cell migration and invasion [50] and regulating the putative tumor suppressor gene *ID4* [51]. Low LRCH1 levels, which increase migration of CD4+ T cells, have also been found in patients with ulcerative colitis [52]. Moreover, Cdc42 is implicated in Natural Killer (NK) cell cytotoxicity: Wiskott-Aldrich Syndrome protein which is the effector of Cdc42 is required for NK cell killing activity [53]. Experimental evidence has shown that LRCH1 may regulate NK-92 cell cytotoxicity [54]. Of further relevance to our finding, Cdc42 is implicated in insulin secretion and is linked to insulin resistance and diabetic nephropathy [55]. One of the proposed mechanisms is via the Cdc42-p21-activated kinase1 (PAK1) signaling pathway essential for insulin secretion in human islets, as it was shown that individuals with diabetes were more likely to have an abnormal component of PAK1 [56]. These data demonstrate a link between *LRCH1* and immune function via Cdc42 that is related to colorectal cancer and diabetes, which may explain the observed differential association. However, functional follow-up studies are needed to further explore this potential significant finding.

To our knowledge, there has been one previous study examining the interaction between T2D genetic variants and diabetes status in colorectal cancer risk, which included 1798 colorectal cancer cases and 1810 controls and focused on T2D-related variants [7]. That study found a statistically significant interaction of T2D with an intronic variant rs4402960 located at the *IGF2BP2* gene (interaction *P*-value: 0.040) and a missense variant rs1801282 at the *PPARG* gene (interaction *P*-value: 0.036) The respective *p*-values for interaction for rs4402960 and rs1801282 with diabetes on colorectal cancer were not nominally significant in our GxE analysis providing limited support for those previously observed interactions. Additionally, we previously conducted an analysis among a large subset of our studies (26,017 cases and 20,692 controls) evaluating interactions between genetic predicted gene-expression levels and diabetes on colorectal cancer risk, and identified a statistically significant interaction between genetically predicted gene expression levels for *PTPN2* and diabetes (*P*-value: 2.31 × 10^−5^) [57]. As the approach of this previous analysis was use of multiple common variants to predict gene expression, we would not expect to replicate those findings here.

Strengths of this study include the large sample size and state of the art statistical approaches, including 2-step [16] and joint tests [17, 22, 23], that improved statistical power by leveraging direct gene-diabetes and gene-colorectal cancer associations induced by Gxdiabetes effects on colorectal cancer risk. We applied strict corrections to account for multiple comparisons because of the number of the methods used. For the two novel variants we identified, we performed sensitivity analyses additionally adjusting our models for BMI, GxBMI, and BMIxDiabetes. In these adjusted models, the GxDiabetes effect estimate changed very little (less than 0.2%) and both interactions remained statistically significant. We acknowledge that our results were limited to European descent individuals and thus our findings cannot be readily generalized to other populations but require follow up in those population groups where GxE efforts are underpowered. Additional harmonization of epidemiological data is ongoing and as such we will expand GxE testing once this is complete. We used self-reported diabetes to define our exposure which may be subject to measurement error in the traditional case-control settings. However, measurement and imputation of G should be non-differential with respect to both diabetes and colorectal cancer status. Thus, while measurement error may lead to reduced power to detect GxE interaction, we do not expect it to lead to spurious associations if G and E are independent. In addition, our novel findings need to be explored in experimental models. Also, we could not account for diabetes history and treatment which may have an effect on colorectal cancer risk. For example, an inverse association between metformin use and colorectal cancer risk has been found in some studies, but not all, while a clinical trial conducted in Japan reported a protective effect of metformin on colorectal polyp development [58]. Future studies may also focus on incorporating data on pre-diabetes states and those with hyperinsulinemia.

In summary, our results suggest that variation in genes related to immune function and regulation of the insulin receptor and PI3K activity may modify the association between diabetes and colorectal cancer risk. These results provide novel insights into the biology underlying diabetes and colorectal cancer relationship. Further experimental studies are warranted to understand the mechanisms by which these genes play a role in linking diabetes and colorectal cancer development.

## Supplementary information

STROBE checklist

Supplementary Methods and Figures

Supplementary Tables

## Data Availability

The data underlying this article will be deposited into a public repository and the accession codes will be available before publication.

## Code availability

The code used to generate results of this article will be shared on reasonable request to the corresponding author.
