# Supplementary material for: Probing the diabetes and colorectal cancer relationship using gene – environment interaction analyses
Source: Br J Cancer. 2023 Jun 26;129(3):511–20. doi: 10.1038/s41416-023-02312-z (PMC10403521; doi:10.1038/s41416-023-02312-z)
Supplement: Supplementary file 3 — Supplementary Tables [file 41416_2023_2312_MOESM3_ESM.docx]

| **Supplementary Table S1**. Description of studies included in the gene-diabetes interaction analysis for colorectal cancer risk. | | | | | | | | | | |
| --- | --- | --- | --- | --- | --- | --- | --- | --- | --- | --- |
|  |  |  |  |  | **Colorectal cancer cases (N=31,318)** | | | **Controls (N=41,499)** | | |
| **Study Acronym** | **Study Name** | **Study Design** | **Country** | **Genotyping Platform** | **Individuals with diabetes (%)** | **Individuals without diabetes (%)** | **Total** | **Individuals with diabetes (%)** | **Individuals without diabetes (%)** | **Total** |
| ASTERISK | Association STudy Evaluating RISK for sporadic colorectal cancer | case-control | France | Illumina 300K | 101 (11.4) | 784 (88.6) | 885 | 47 (5.0) | 897 (95.0) | 944 |
| CCFR_1 | Colon Cancer Family Registry | case- control | USA, Canada, Australia | Illumina 1M, 1M duo | 88 (8.6) | 930 (91.4) | 1018 | 85 (8.8) | 882 (91.2) | 967 |
| CCFR_3 | Colon Cancer Family Registry | case- control | USA, Canada, Australia | Affymetrix Axiom | 84 (9.8) | 771 (90.2) | 855 | 66 (10.4) | 570 (89.6) | 636 |
| CCFR_4 | Colon Cancer Family Registry | case- control | USA, Canada, Australia | Illumina Oncoarray | 140 (12.0) | 1,024 (88.0) | 1,164 | 31 (4.8) | 617 (95.2) | 648 |
| CLUEII | Campaign against Cancer and Heart Disease II | cohort | USA | Illumina Oncoarray+ custom iSelect | 16 (6.0) | 249 (94.0) | 265 | 11 (4.4) | 241 (95.6) | 252 |
| Colo23 | Hawaii Colorectal Cancer Studies 2&3 | case- series | USA | Illumina 300K | 6 (6.9) | 81 (93.1) | 87 | 7 (5.7) | 116 (94.3) | 123 |
| CORSA_1 | Colorectal Cancer Study of Austria | case-control | Austria | Affymetrix Axiom | 169 (14.1) | 1,029 (85.9) | 1,198 | 68 (17.5) | 320 (82.5) | 388 |
| CORSA_2 | Colorectal Cancer Study of Austria | case-control | Austria | Illumina Oncoarray+ custom iSelect | 180 (19.4) | 747 (80.6) | 927 | 159 (15.5) | 864 (84.5) | 1023 |
| CPSII_1 | American Cancer Society Cancer Prevention Study II nested case-control study | cohort | USA | Affymetrix Axiom | 48 (8.9) | 493 (91.1) | 541 | 40 (7.5) | 496 (92.5) | 536 |
| CPSII_2 | American Cancer Society Cancer Prevention Study II nested case-control study | cohort | USA | Illumina Oncoarray+ custom iSelect | 27 (7.9) | 313 (92.1) | 340 | 18 (5.2) | 329 (94.8) | 347 |
| CRCGEN | Colorectal Cancer Genetics & Genomics, Spanish study | case- control | Spain | Illumina Oncoarray | 128 (14.9) | 732 (85.1) | 860 | 129 (12.7) | 885 (87.3) | 1,014 |
| CzechCCS | Czech Republic CCS | case- control | Czech Republic | Illumina Oncoarray+ custom iSelect | 288 (21.1) | 1,078 (78.9) | 1,366 | 97 (11.1) | 776 (88.9) | 873 |
| DACHS_1 | Darmkrebs: Chancen der Verhütung durch Screening | case- control | Germany | Illumina 300K | 297 (18.2) | 1,338 (81.8) | 1,635 | 231 (13.8) | 1,441 (86.2) | 1,672 |
| DACHS_2 | Darmkrebs: Chancen der Verhütung durch Screening | case- control | Germany | Illumina OmniExpress | 125 (19.3) | 522 (80.7) | 647 | 75 (15.5) | 410 (84.5) | 485 |
| DACHS_3 | Darmkrebs: Chancen der Verhütung durch Screening Study | case- control | Germany | Illumina OmniExpressExome | 244 (20.2) | 962 (79.8) | 1,206 | 72 (11.7) | 545 (88.3) | 617 |
| EPIC | European Prospective Investigation into Cancer and Nutrition | cohort | Europe | Illumina OmniExpressExome | 103 (5.2) | 1,896 (94.8) | 1,999 | 85 (3.7) | 2,206 (96.3) | 2,291 |
| ESTHER_VERDI | Epidemiologische Studie zu Chancen der Verhutung, Fruherkennung und optimierten THerapie chronischer ERkrankungen in der alteren Bevolkerungstudy | case-control | Germany | Illumina Oncoarray | 53 (13) | 355 (87) | 408 | 51 (12.3) | 363 (87.7) | 414 |
| HawaiiCCS_AD | Hawaii Adenoma Study | case- control | USA | Illumina Oncoarray+ custom iSelect | 9 (11.4) | 70 (88.6) | 79 | 29 (5.5) | 500 (94.5) | 529 |
| HPFS_1_2 | Health Professionals Follow-Up Study | cohort | USA | Illumina OmniExpress | 7 (3.6) | 190 (96.4) | 197 | 3 (1.2) | 250 (98.8) | 253 |
| HPFS_3_AD | Health Professionals Follow-Up Study | cohort | USA | Illumina OmniExpress | 1 (0.3) | 308 (99.7) | 309 | 2 (0.7) | 286 (99.3) | 288 |
| HPFS_4 | Health Professionals Follow-Up Study | cohort | USA | Illumina OmniExpressExome | 26 (14.2) | 157 (85.8) | 183 | 13 (6.6) | 185 (93.4) | 198 |
| HPFS_5_AD | Health Professionals Follow-Up Study | cohort | USA | Illumina Oncoarray+custom iSelect | 21 (13.5) | 134 (86.5) | 155 | 5 (4.8) | 100 (95.2) | 105 |
| Kentucky | Kentucky Case-Control Study | case- control | USA | Affymetrix Axiom | 196 (22.8) | 662 (77.2) | 858 | 190 (18.4) | 844 (81.6) | 1,034 |
| LCCS | Leeds Colorectal Cancer Study | case- control | UK | Illumina Oncoarray+ custom iSelect | 159 (11.4) | 1,234 (88.6) | 1,393 | 53 (7.8) | 628 (92.2) | 681 |
| MCCS_1 | Melbourne Collaborative Cohort Study | cohort | Australia | Affymetrix Axiom | 22 (4.1) | 512 (95.9) | 534 | 12 (2.6) | 449 (97.4) | 461 |
| MCCS_2 | Melbourne Collaborative Cohort Study | cohort | Australia | Affymetrix Axiom | 8 (3.7) | 210 (96.3) | 218 | 4 (1.9) | 208 (98.1) | 212 |
| MEC_1 | Multiethnic Cohort Study | cohort | USA | Illumina 300K | 20 (6.2) | 301 (93.8) | 321 | 18 (5.3) | 322 (94.7) | 340 |
| NCCCSI | North Carolina Colon Cancer Study, I | case- control | USA | Illumina Oncoarray+ custom iSelect | 48 (19.9) | 193 (80.1) | 241 | 62 (13.4) | 401 (86.6) | 463 |
| NCCCSII | North Carolina Colon Cancer Study, II | case- control | USA | Illumina Oncoarray+ custom iSelect | 96 (16.6) | 483 (83.4) | 579 | 89 (13.2) | 584 (86.8) | 673 |
| NFCCR_2 | Newfoundland Case-Control Study | case- control | Canada | Affymetrix Axiom | 45 (23.4) | 147 (76.6) | 192 | 65 (14) | 400 (86) | 465 |
| NHS_1_2 | Nurses Health Study | cohort | USA | Illumina OmniExpress | 17 (4.6) | 356 (95.4) | 373 | 19 (2.5) | 751 (97.5) | 770 |
| NHS_3_AD | Nurses Health Study | cohort | USA | Illumina OmniExpress | 11 (2.2) | 489 (97.8) | 500 | 6 (1.4) | 410 (98.6) | 416 |
| NHS_4 | Nurses Health Study | cohort | USA | Illumina OmniExpressExome | 41 (13.8) | 257 (86.2) | 298 | 29 (9.6) | 272 (90.4) | 301 |
| NHS_5_AD | Nurses Health Study | cohort | USA | Illumina Oncoarray+ custom iSelect | 30 (12.1) | 217 (87.9) | 247 | 8 (3.7) | 210 (96.3) | 218 |
| PHS | Physician’s Health Study | cohort | USA | Illumina OmniExpress | 10 (3.2) | 299 (96.8) | 309 | 12 (3.1) | 373 (96.9) | 385 |
| PLCO_1_Rematch | Prostate, Lung, Colorectal, and Ovarian Cancer Screening Trial | cohort | USA | Illumina 300/240S & 610K | 50 (9.4) | 480 (90.6) | 530 | 27 (8.4) | 296 (91.6) | 323 |
| PLCO_2 | Prostate, Lung, Colorectal, and Ovarian Cancer Screening Trial | cohort | USA | Illumina 300K | 39 (8.3) | 429 (91.7) | 468 | 20 (5.5) | 346 (94.5) | 366 |
| PLCO_3 | Prostate, Lung, Colorectal, and Ovarian Cancer Screening Trial | cohort | USA | Illumina 300K | 46 (6.2) | 693 (93.8) | 739 | 204 (6.0) | 3,180 (94.0) | 3,384 |
| PLCO_4_AD | Prostate, Lung, Colorectal, and Ovarian Cancer Screening Trial | cohort | USA | Illumina Oncoarray+ custom iSelect | 80 (7.3) | 1019 (92.7) | 1099 | 65 (7.0) | 863 (93.0) | 928 |
| REACH_AD | Colon Cancer Pathways: Hyperplastic Polyps and Adenomas | case-control | USA | Illumina Oncoarray+ custom iSelect | 12 (15.4) | 66 (84.6) | 78 | 28 (9.0) | 283 (91.0) | 311 |
| SELECT | Selenium and Vitamin E Prevention Trial | clinical trial | USA | Illumina Oncoarray+ custom iSelect | 17 (6.5) | 245 (93.5) | 262 | 29 (10.8) | 239 (89.2) | 268 |
| SMC_COSM | Swedish Mammography Cohort and Cohort of Swedish Men | cohort | Sweden | Illumina Oncoarray | 108 (18.7) | 470 (81.3) | 578 | 118 (13.8) | 738 (86.2) | 856 |
| SMS_AD | Screening Markers for Colorectal Cancer Study (advanced adenomas) | case- control | USA | Illumina Oncoarray+ custom iSelect | 5 (12.2) | 36 (87.8) | 41 | 15 (11.8) | 112 (88.2) | 127 |
| UKB_1 | UK Biobank | cohort | UK | UK Biobank Axiom | 207 (7.1) | 2,704 (92.9) | 2,911 | 720 (6.2) | 10,871 (93.8) | 11,591 |
| VITAL | VITamins And Lifestyle | cohort | USA | Illumina 300K | 10 (3.7) | 260 (96.3) | 270 | 2 (0.7) | 280 (99.3) | 282 |
| WHI_1 | Womens Health Initiative Study | cohort | USA | Illumina 550K, 550Kduo, 610K | 33 (7.3) | 416 (92.7) | 449 | 18 (3.5) | 497 (96.5) | 515 |
| WHI_2 | Womens Health Initiative Study | cohort | USA | Illumina 300K | 70 (7.3) | 892 (92.7) | 962 | 51 (5.2) | 923 (94.8) | 974 |
| WHI_3 | Womens Health Initiative Study | cohort | USA | Illumina Oncoarray+ custom iSelect | 17 (3.1) | 527 (96.9) | 544 | 18 (3.3) | 534 (96.7) | 552 |
| **Total** |  |  |  |  | **3,558** | **27,760** | **31,318** | **3,206** | **38,293** | **41,499** |

| **Supplementary Table S2.** Dataset sources for chromatin accessibility analysis. | | | |
| --- | --- | --- | --- |
| Assay | Dataset | N Bio Replicates | Accession |
| DNASE-seq | Healthy controls | 3 | GEO: GSM2058015, GSM2058016, GSM2058017 |
| DNASE-seq | Primary tumor samples | 3 | GEO: GSM2058018, GSM2058019, GSM2058020 |
| DNASE-seq | HCT116 | 1 | ENCODE: ENCSR000ENM |
| DNASE-seq | COLO205 | 1 | GEO: GSM2224588 |
| DNASE-seq | SW480 | 1 | ENCODE: ENCSR217SET |
| H3K27ac histone ChIP-seq | Healthy controls | 4 | GEO: GSM2058021, GSM2058022, GSM2058023, GSM2058024 |
| H3K27ac histone ChIP-seq | Tumor | 24 | GEO: GSM2058028, GSM2058029, GSM2058030, GSM2058031, GSM2058032, GSM2058033, GSM2058034, GSM2058035, GSM2058036, GSM2058037, GSM2058038, GSM2058039, GSM2058040, GSM2058041, GSM2058042, GSM2058043, GSM2058044, GSM2058045, GSM2058046, GSM2058047, GSM2058048, GSM2058049, GSM2058050, GSM2058051, GSM2058052, GSM2058053, GSM2058054, GSM2058055, GSM2058056 |
| H3K27ac histone ChIP-seq | HCT116 | 1 | GEO: GSM2058026 |
| H3K27ac histone ChIP-seq | COLO205 | 1 | GEO: GSM2058025 |
| H3K27ac histone ChIP-seq | SW480 | 1 | GEO: GSM2058027 |
| H3K4me1 histone ChIP-seq | Healthy controls | 4 | GEO: GSM2058059, GSM2058060, GSM2058061, GSM2058062 |
| H3K4me1 histone ChIP-seq | Tumor | 24 | GEO: GSM2058066, GSM2058067, GSM2058068, GSM2058069, GSM2058070, GSM2058071, GSM2058072, GSM2058073, GSM2058074, GSM2058075, GSM2058076, GSM2058077, GSM2058078, GSM2058079, GSM2058080, GSM2058081, GSM2058082, GSM2058083, GSM2058084, GSM2058085, GSM2058086, GSM2058087, GSM2058088, GSM2058089 |
| H3K4me1 histone ChIP-seq | HCT116 | 1 | GEO: GSM2058064 |
| H3K4me1 histone ChIP-seq | COLO205 | 1 | GEO: GSM2058063 |
| H3K4me1 histone ChIP-seq | SW480 | 1 | GEO: GSM2058065 |

| **Supplementary Table S3:** Composition of training, validation, and test sets for hg19 for 10-fold cross-validation of support vector machines. | | | |
| --- | --- | --- | --- |
| Fold | Training chromosomes | Validation chromosomes | Test chromosomes |
| 1 | chr2, chr3, chr4, chr5, chr6, chr7, chr9, chr11, chr12, chr13, chr14, chr15, chr16, chr17, chr18, chr19, chr20, chr21, chr22, chrX, chrY | chr8, chr10 | chr1 |
| 2 | chr3, chr4, chr5, chr6, chr7, chr8, chr9, chr10, chr11, chr12, chr13, chr14, chr15, chr16, chr17, chr18, chr20, chr21, chr22, chrX, chrY | chr1 | chr19, chr10 |
| 3 | chr1, chr4, chr5, chr6, chr7, chr8, chr9, chr10, chr11, chr12, chr13, chr14, chr15, chr16, chr17, chr18, chr21, chr22, chrX, chrY | chr19, chr2 | chr3, chr20 |
| 4 | chr1, chr2, chr4, chr5, chr7, chr8, chr9, chr10, chr11, chr12, chr14, chr15, chr16, chr17, chr18, chr19, chr21, chrX, chrY | chr3, chr20 | chr13, chr6, chr22 |
| 5 | chr1, chr2, chr3, chr4, chr7, chr8, chr9, chr10, chr11, chr12, chr14, chr15, chr17, chr18, chr19, chr20, chr21, chrX | chr13, chr6, chr22 | chr5,chr16, chrY |
| 6 | chr1, chr2, chr3, chr6, chr7, chr8, chr9, chr10, chr11, chr12, chr13, chr14, chr17, chr18, chr19, chr20, chr22, chrX | chr5, chr16, chrY | chr4, chr15, chr21 |
| 7 | chr1, chr2, chr3, chr5, chr6, chr8, chr9, chr10, chr11, chr12, chr13, chr16, chr17, chr19, chr20, chr22, chrX, chrY | chr4, chr15, chr21 | chr7, chr18, chr14 |
| 8 | chr1, chr2, chr3, chr4, chr5, chr6, chr8, chr9, chr10, chr12, chr13, chr15, chr16, chr19, chr20, chr21, chr22, chrY | chr7, chr18, chr14 | chr11, chr17, chrX |
| 9 | chr1, chr2, chr3, chr4, chr5, chr6, chr7, chr8, chr10, chr13, chr14, chr15, chr16, chr18, chr19, chr20, chr21, chr22, chrY | chr11, chr17, chrX | chr12, chr9 |
| 10 | chr1, chr2, chr3, chr4, chr5, chr6, chr7, chr11, chr13, chr14, chr15, chr16, chr17, chr18, chr19, chr20, chr21, chr22, chrX, chrY | chr12, chr9 | chr10, chr8 |

| **Supplementary Table 4.** eQTL results from normal colon tissue in BarcUVa-Seq data for variant rs3802177 and rs9526201 regions (and variants in *R*^2^>0.5 with rs9526201 and rs3802177). | | | | | | | | |
| --- | --- | --- | --- | --- | --- | --- | --- | --- |
| rsid | Chromosome | Position (hg19) | Reference allele | Alternate allele | Ensembl_gene_id | Gene | Beta | P-value |
| rs3802177 region | | | | | | | | |
| rs3802177 | 8 | 118185025 | G | A | ENSG00000205002 | *AARD* | -0.14 | 4.72E-02 |
| rs13266634 | 8 | 118184783 | C | T | ENSG00000205002 | *AARD* | -0.14 | 4.76E-02 |
| rs11774700 | 8 | 118220270 | T | C | ENSG00000205002 | *AARD* | -0.18 | 1.84E-02 |
| rs9526201 region | | | | | | | | |
| rs4941561 | 13 | 47118651 | C | T | ENSG00000102445 | *RUBCNL* | 0.2 | 1.61E-03 |
| rs1012628 | 13 | 47119642 | C | T | ENSG00000102445 | *RUBCNL* | 0.2 | 1.61E-03 |
| rs2405413 | 13 | 47113763 | T | C | ENSG00000102445 | *RUBCNL* | 0.2 | 1.73E-03 |
| rs7994252 | 13 | 47115608 | A | G | ENSG00000102445 | *RUBCNL* | 0.2 | 1.78E-03 |
| rs4941563 | 13 | 47121510 | C | A | ENSG00000102445 | *RUBCNL* | 0.19 | 2.09E-03 |
| rs9534425 | 13 | 47126767 | G | A | ENSG00000102445 | *RUBCNL* | 0.18 | 3.69E-03 |
| rs7319949 | 13 | 47148843 | T | G | ENSG00000102445 | *RUBCNL* | 0.18 | 5.69E-03 |
| rs1886219 | 13 | 47159881 | A | T | ENSG00000102445 | *RUBCNL* | 0.17 | 5.92E-03 |
| rs1886220 | 13 | 47159484 | A | G | ENSG00000102445 | *RUBCNL* | 0.17 | 5.93E-03 |
| rs1535792 | 13 | 47165040 | T | A | ENSG00000102445 | *RUBCNL* | 0.17 | 6.08E-03 |
| rs7337752 | 13 | 47156855 | T | C | ENSG00000102445 | *RUBCNL* | 0.17 | 6.40E-03 |
| rs4942557 | 13 | 47183068 | G | A | ENSG00000102445 | *RUBCNL* | 0.17 | 6.66E-03 |
| rs6561323 | 13 | 47185601 | A | G | ENSG00000102445 | *RUBCNL* | 0.17 | 6.69E-03 |
| rs4942558 | 13 | 47206582 | T | C | ENSG00000102445 | *RUBCNL* | 0.17 | 6.80E-03 |
| rs7986104 | 13 | 47206196 | C | G | ENSG00000102445 | *RUBCNL* | 0.17 | 6.80E-03 |
| rs4942559 | 13 | 47206814 | G | C | ENSG00000102445 | *RUBCNL* | 0.17 | 6.81E-03 |
| rs7318773 | 13 | 47208947 | G | T | ENSG00000102445 | *RUBCNL* | 0.17 | 6.81E-03 |
| rs9526208 | 13 | 47208767 | A | G | ENSG00000102445 | *RUBCNL* | 0.17 | 6.84E-03 |
| rs842406 | 13 | 47230194 | C | T | ENSG00000102445 | *RUBCNL* | 0.17 | 6.85E-03 |
| rs912434 | 13 | 47189928 | G | T | ENSG00000102445 | *RUBCNL* | 0.17 | 6.86E-03 |
| rs7995870 | 13 | 47172275 | T | G | ENSG00000102445 | *RUBCNL* | 0.19 | 7.12E-03 |
| rs1570620 | 13 | 47201874 | C | T | ENSG00000102445 | *RUBCNL* | 0.16 | 7.17E-03 |
| rs9526207 | 13 | 47201226 | C | T | ENSG00000102445 | *RUBCNL* | 0.16 | 7.17E-03 |
| rs2181786 | 13 | 47199611 | T | C | ENSG00000102445 | *RUBCNL* | 0.16 | 7.17E-03 |
| rs4942555 | 13 | 47174469 | A | G | ENSG00000102445 | *RUBCNL* | 0.18 | 7.91E-03 |
| rs4942556 | 13 | 47174585 | C | T | ENSG00000102445 | *RUBCNL* | 0.18 | 7.95E-03 |
| rs1886964 | 13 | 47175084 | C | T | ENSG00000102445 | *RUBCNL* | 0.18 | 8.01E-03 |
| rs9316217 | 13 | 47209806 | G | C | ENSG00000102445 | *RUBCNL* | 0.16 | 8.08E-03 |
| rs1467605 | 13 | 47211861 | A | C | ENSG00000102445 | *RUBCNL* | 0.16 | 8.13E-03 |
| rs9534447 | 13 | 47212722 | G | C | ENSG00000102445 | *RUBCNL* | 0.16 | 8.14E-03 |
| rs9534448 | 13 | 47212961 | G | A | ENSG00000102445 | *RUBCNL* | 0.16 | 8.16E-03 |
| rs4942566 | 13 | 47216011 | G | C | ENSG00000102445 | *RUBCNL* | 0.16 | 8.23E-03 |
| rs1773118 | 13 | 47221640 | T | G | ENSG00000102445 | *RUBCNL* | 0.16 | 8.33E-03 |
| rs1216862 | 13 | 47232678 | A | G | ENSG00000102445 | *RUBCNL* | 0.16 | 9.33E-03 |
| rs842370 | 13 | 47238854 | A | G | ENSG00000102445 | *RUBCNL* | 0.16 | 9.40E-03 |
| rs1773122 | 13 | 47222433 | A | G | ENSG00000102445 | *RUBCNL* | 0.16 | 1.02E-02 |
| rs9316222 | 13 | 47214690 | T | C | ENSG00000102445 | *RUBCNL* | 0.16 | 1.03E-02 |
| rs1467604 | 13 | 47211879 | G | A | ENSG00000102445 | *RUBCNL* | 0.15 | 1.11E-02 |
| rs1408223 | 13 | 47215674 | A | G | ENSG00000102445 | *RUBCNL* | 0.15 | 1.11E-02 |
| rs842407 | 13 | 47228730 | C | G | ENSG00000102445 | *RUBCNL* | 0.16 | 1.13E-02 |
| rs9526212 | 13 | 47225745 | A | G | ENSG00000102445 | *RUBCNL* | 0.15 | 1.17E-02 |
| rs7335684 | 13 | 47193696 | G | A | ENSG00000102445 | *RUBCNL* | 0.15 | 1.19E-02 |
| rs1773124 | 13 | 47223835 | G | A | ENSG00000102445 | *RUBCNL* | 0.15 | 1.26E-02 |
| rs9526201 | 13 | 47191972 | G | A | ENSG00000102445 | *RUBCNL* | 0.17 | 1.34E-02 |
| rs9534439 | 13 | 47192049 | T | C | ENSG00000102445 | *RUBCNL* | 0.17 | 1.41E-02 |
| rs1535791 | 13 | 47165458 | T | C | ENSG00000102445 | *RUBCNL* | 0.15 | 1.58E-02 |
| rs1123028 | 13 | 47166195 | C | T | ENSG00000102445 | *RUBCNL* | 0.15 | 1.65E-02 |
| rs7328294 | 13 | 47159296 | T | A | ENSG00000102445 | *RUBCNL* | 0.15 | 1.66E-02 |
| rs9316214 | 13 | 47157872 | C | T | ENSG00000102445 | *RUBCNL* | 0.15 | 1.66E-02 |
| rs9534433 | 13 | 47158041 | G | A | ENSG00000102445 | *RUBCNL* | 0.15 | 1.67E-02 |
| rs9534432 | 13 | 47157969 | C | T | ENSG00000102445 | *RUBCNL* | 0.15 | 1.67E-02 |
| rs6561321 | 13 | 47161560 | C | A | ENSG00000102445 | *RUBCNL* | 0.15 | 1.80E-02 |
| rs1570621 | 13 | 47170118 | T | C | ENSG00000102445 | *RUBCNL* | 0.19 | 1.86E-02 |
| rs912428 | 13 | 47167903 | A | G | ENSG00000102445 | *RUBCNL* | 0.16 | 2.08E-02 |
| rs7330453 | 13 | 47171755 | G | A | ENSG00000102445 | *RUBCNL* | 0.16 | 2.17E-02 |
| rs1570622 | 13 | 47169092 | G | A | ENSG00000102445 | *RUBCNL* | 0.15 | 2.29E-02 |
| rs912427 | 13 | 47169273 | T | C | ENSG00000102445 | *RUBCNL* | 0.15 | 2.30E-02 |
| rs912426 | 13 | 47169419 | C | T | ENSG00000102445 | *RUBCNL* | 0.15 | 2.31E-02 |
| rs6561322 | 13 | 47163185 | T | A | ENSG00000102445 | *RUBCNL* | 0.15 | 2.32E-02 |
| rs9567707 | 13 | 47175435 | T | C | ENSG00000102445 | *RUBCNL* | 0.15 | 2.49E-02 |
| rs4942561 | 13 | 47209347 | G | T | ENSG00000102445 | *RUBCNL* | 0.13 | 2.52E-02 |
| rs1535793 | 13 | 47154966 | G | A | ENSG00000102445 | *RUBCNL* | 0.14 | 2.89E-02 |
| rs1747223 | 13 | 47224681 | A | G | ENSG00000102445 | *RUBCNL* | 0.13 | 3.02E-02 |
| rs4942553 | 13 | 47155975 | A | G | ENSG00000102445 | *RUBCNL* | 0.13 | 3.05E-02 |
| rs1007171 | 13 | 47106226 | T | G | ENSG00000102445 | *RUBCNL* | 0.15 | 3.54E-02 |
| rs7994173 | 13 | 47107188 | A | T | ENSG00000102445 | *RUBCNL* | 0.15 | 3.69E-02 |
| rs4942550 | 13 | 47104680 | A | G | ENSG00000102445 | *RUBCNL* | 0.15 | 3.71E-02 |
| rs1570974 | 13 | 47108253 | G | A | ENSG00000102445 | *RUBCNL* | 0.15 | 3.74E-02 |
| rs1570975 | 13 | 47103489 | T | C | ENSG00000102445 | *RUBCNL* | 0.15 | 3.77E-02 |
| rs7333028 | 13 | 47238684 | T | C | ENSG00000102445 | *RUBCNL* | 0.13 | 3.78E-02 |
| rs9534454 | 13 | 47234659 | T | C | ENSG00000102445 | *RUBCNL* | 0.13 | 3.85E-02 |
| rs9526214 | 13 | 47237213 | T | C | ENSG00000102445 | *RUBCNL* | 0.13 | 3.93E-02 |
| rs1326113 | 13 | 47100585 | T | C | ENSG00000102445 | *RUBCNL* | 0.15 | 4.03E-02 |
| rs1326112 | 13 | 47100498 | C | T | ENSG00000102445 | *RUBCNL* | 0.15 | 4.04E-02 |
| rs1326111 | 13 | 47100405 | T | C | ENSG00000102445 | *RUBCNL* | 0.15 | 4.04E-02 |
| rs4942549 | 13 | 47099664 | C | T | ENSG00000102445 | *RUBCNL* | 0.15 | 4.09E-02 |
| rs6561316 | 13 | 47099561 | A | T | ENSG00000102445 | *RUBCNL* | 0.15 | 4.10E-02 |
| rs6561315 | 13 | 47099426 | T | G | ENSG00000102445 | *RUBCNL* | 0.15 | 4.10E-02 |
| rs6561314 | 13 | 47099042 | C | T | ENSG00000102445 | *RUBCNL* | 0.15 | 4.13E-02 |
| rs6561313 | 13 | 47098897 | C | A | ENSG00000102445 | *RUBCNL* | 0.15 | 4.13E-02 |
| rs6561312 | 13 | 47098806 | G | A | ENSG00000102445 | *RUBCNL* | 0.15 | 4.15E-02 |
| rs1886967 | 13 | 47098648 | A | T | ENSG00000102445 | *RUBCNL* | 0.15 | 4.15E-02 |
| rs7330387 | 13 | 47097802 | T | C | ENSG00000102445 | *RUBCNL* | 0.15 | 4.17E-02 |
| rs7328711 | 13 | 47097643 | C | T | ENSG00000102445 | *RUBCNL* | 0.15 | 4.18E-02 |
| rs4942548 | 13 | 47097278 | G | C | ENSG00000102445 | *RUBCNL* | 0.15 | 4.21E-02 |
| rs4942547 | 13 | 47097227 | C | T | ENSG00000102445 | *RUBCNL* | 0.15 | 4.22E-02 |
| rs9526189 | 13 | 47097111 | C | T | ENSG00000102445 | *RUBCNL* | 0.15 | 4.22E-02 |
| rs4942546 | 13 | 47097226 | A | G | ENSG00000102445 | *RUBCNL* | 0.15 | 4.22E-02 |
| rs7317158 | 13 | 47096173 | G | T | ENSG00000102445 | *RUBCNL* | 0.15 | 4.23E-02 |
| rs9534416 | 13 | 47096271 | C | T | ENSG00000102445 | *RUBCNL* | 0.15 | 4.23E-02 |
| rs9526188 | 13 | 47096658 | C | T | ENSG00000102445 | *RUBCNL* | 0.15 | 4.23E-02 |
| rs7139769 | 13 | 47095886 | G | A | ENSG00000102445 | *RUBCNL* | 0.15 | 4.23E-02 |
| rs4942545 | 13 | 47095594 | G | A | ENSG00000102445 | *RUBCNL* | 0.15 | 4.23E-02 |

| **Supplementary Table 5.** eQTL results from GTEx transverse colon tissue data for variants within rs3802177 and rs9526201 regions (and variants in *R*^2^>0.5 with rs3802177 and rs9526201). | | | | | | | | |
| --- | --- | --- | --- | --- | --- | --- | --- | --- |
| rsid | Chromosome | Position (hg38) | Reference allele | Alternate allele | Ensembl_gene_id | Gene | Slope (*β*) | P-value |
| rs3802177 region | | | | | | | | |
| rs11774700 | 8 | 117208031 | T | C | ENSG00000205002 | *AARD* | 0.08 | 1.77E-02 |
| rs4300038 | 8 | 117205676 | G | A | ENSG00000205002 | *AARD* | 0.07 | 3.37E-02 |
| rs11558471 | 8 | 117173494 | A | G | ENSG00000205002 | *AARD* | 0.08 | 2.10E-02 |
| rs13266634 | 8 | 117172544 | C | T | ENSG00000205002 | *AARD* | 0.09 | 1.42E-02 |
| rs11774700 | 8 | 117208031 | T | C | ENSG00000205002 | *AARD* | 0.08 | 1.77E-02 |
| rs4300038 | 8 | 117205676 | G | A | ENSG00000205002 | *AARD* | 0.07 | 3.37E-02 |
| rs11558471 | 8 | 117173494 | A | G | ENSG00000205002 | *AARD* | 0.08 | 2.10E-02 |
| rs13266634 | 8 | 117172544 | C | T | ENSG00000205002 | *AARD* | 0.09 | 1.42E-02 |
| rs9526201 region | | | | | | | | |
| rs7996004 | 13 | 46519367 | G | T | ENSG00000136141 | *LRCH1* | -0.08 | 1.59E-02 |
| rs1570974 | 13 | 46534118 | G | A | ENSG00000136141 | *LRCH1* | -0.08 | 1.64E-02 |
| rs7328711 | 13 | 46523508 | C | T | ENSG00000136141 | *LRCH1* | -0.08 | 1.75E-02 |
| rs6561312 | 13 | 46524671 | G | A | ENSG00000136141 | *LRCH1* | -0.08 | 1.75E-02 |
| rs6561315 | 13 | 46525291 | T | G | ENSG00000136141 | *LRCH1* | -0.08 | 1.75E-02 |
| rs1326111 | 13 | 46526270 | T | C | ENSG00000136141 | *LRCH1* | -0.08 | 1.75E-02 |
| rs7317158 | 13 | 46522038 | G | T | ENSG00000136141 | *LRCH1* | -0.08 | 1.75E-02 |
| rs9526189 | 13 | 46522976 | C | T | ENSG00000136141 | *LRCH1* | -0.08 | 1.75E-02 |
| rs4942548 | 13 | 46523143 | G | C | ENSG00000136141 | *LRCH1* | -0.08 | 1.75E-02 |
| rs1886967 | 13 | 46524513 | A | T | ENSG00000136141 | *LRCH1* | -0.08 | 1.75E-02 |
| rs6561314 | 13 | 46524907 | C | T | ENSG00000136141 | *LRCH1* | -0.08 | 1.75E-02 |
| rs7994173 | 13 | 46533053 | A | T | ENSG00000136141 | *LRCH1* | -0.08 | 1.75E-02 |
| rs4942549 | 13 | 46525529 | C | T | ENSG00000136141 | *LRCH1* | -0.08 | 1.75E-02 |
| rs1326112 | 13 | 46526363 | C | T | ENSG00000136141 | *LRCH1* | -0.08 | 1.75E-02 |
| rs7139769 | 13 | 46521751 | G | A | ENSG00000136141 | *LRCH1* | -0.08 | 1.75E-02 |
| rs9526188 | 13 | 46522523 | C | T | ENSG00000136141 | *LRCH1* | -0.08 | 1.75E-02 |
| rs4942547 | 13 | 46523092 | C | T | ENSG00000136141 | *LRCH1* | -0.08 | 1.75E-02 |
| rs35441059 | 13 | 46521015 | T | C | ENSG00000136141 | *LRCH1* | -0.08 | 1.75E-02 |
| rs7330387 | 13 | 46523667 | T | C | ENSG00000136141 | *LRCH1* | -0.08 | 1.75E-02 |
| rs6561313 | 13 | 46524762 | C | A | ENSG00000136141 | *LRCH1* | -0.08 | 1.75E-02 |
| rs6561316 | 13 | 46525426 | A | T | ENSG00000136141 | *LRCH1* | -0.08 | 1.75E-02 |
| rs1326113 | 13 | 46526450 | T | C | ENSG00000136141 | *LRCH1* | -0.08 | 1.75E-02 |
| rs4942545 | 13 | 46521459 | G | A | ENSG00000136141 | *LRCH1* | -0.08 | 1.75E-02 |
| rs9534416 | 13 | 46522136 | C | T | ENSG00000136141 | *LRCH1* | -0.08 | 1.75E-02 |
| rs4942546 | 13 | 46523091 | A | G | ENSG00000136141 | *LRCH1* | -0.08 | 1.75E-02 |
| rs1886966 | 13 | 46520814 | T | C | ENSG00000136141 | *LRCH1* | -0.08 | 1.75E-02 |
| rs71432911 | 13 | 46505522 | A | G | ENSG00000136141 | *LRCH1* | 0.08 | 1.86E-02 |
| rs493582 | 13 | 46501576 | T | C | ENSG00000136141 | *LRCH1* | 0.08 | 1.99E-02 |
| rs4942550 | 13 | 46530545 | A | G | ENSG00000136141 | *LRCH1* | -0.08 | 1.99E-02 |
| rs568025 | 13 | 46499948 | C | A | ENSG00000136141 | *LRCH1* | 0.08 | 1.99E-02 |
| rs643264 | 13 | 46503865 | T | C | ENSG00000136141 | *LRCH1* | 0.08 | 1.99E-02 |
| rs1570975 | 13 | 46529354 | T | C | ENSG00000136141 | *LRCH1* | -0.08 | 1.99E-02 |
| rs642732 | 13 | 46503676 | G | A | ENSG00000136141 | *LRCH1* | 0.08 | 1.99E-02 |
| rs1007171 | 13 | 46532091 | T | G | ENSG00000136141 | *LRCH1* | -0.08 | 1.99E-02 |
| rs4942553 | 13 | 46581840 | A | G | ENSG00000102445 | *RUBCNL* | 0.06 | 4.99E-02 |
| rs1535793 | 13 | 46580831 | G | A | ENSG00000102445 | *RUBCNL* | 0.06 | 4.99E-02 |

| **Supplementary Table 6.** eQTL results from GTEx V8 for rs9526201 genetic variant. | | |
| --- | --- | --- |
| Tissue | Normalized effect size* | P-value |
| heart-left ventricle | -0.29 | 5.4x10^-10^ |
| nerve-tibial | -0.23 | 3.5x10^-8^ |
| whole blood | -0.13 | 4.9x10^-8^ |
| muscle-skeletal | -0.18 | 2.3x10^-7^ |
| adipose–subcutaneous | -0.15 | 5.0x10^-7^ |
| esophagus-muscularis | -0.13 | 1.2x10^-6^ |
| testis | -0.16 | 1.6x10^-5^ |
| artery-aorta | -0.17 | 5.2x10^-5^ |
| **A* vs *G* allele | | |

| **Supplementary Table 7.** Functional annotation results for variant rs9526201 and correlated variants (*R*^2^>0.5) which showed a significant interaction with diabetes in the 2 degrees of freedom gene-environment interaction analyses for colorectal cancer risk. Markers (*R*^2^>0.5 with rs9526201) are marked as “yes” if they coincide with open chromatin regions (the number of cell lines/samples is shown in parenthesis). | | | | | | | | | | | | | | | | | |
| --- | --- | --- | --- | --- | --- | --- | --- | --- | --- | --- | --- | --- | --- | --- | --- | --- | --- |
| rsid | chromosome:position | normal mucosa ATACseq | normal crypt DHS | normal crypt H3K27ac | CRC dhs | CRC H3K27ac | Adrenal or pancreas | CNS | Cardiovascular | Connective or bone | Gastrointestinal | Immune or hematopoietic | Kidney | Liver | Skeletal muscle | Other | *R*^2^ |
| rs9526201 | 13:47191972 | no | no | no | no | yes (6) | no | no | yes | no | yes | yes | no | no | no | yes | 1.000 |
| rs9534439 | 13:47192049 | no | no | no | no | yes (6) | no | no | yes | no | yes | yes | no | no | no | yes | 1.000 |
| rs7995870 | 13:47172275 | no | no | no | no | yes (2) | no | no | yes | no | yes | yes | no | no | yes | yes | 0.957 |
| rs4942555 | 13:47174469 | no | no | no | no | yes (10) | yes | no | no | yes | yes | yes | no | yes | no | yes | 0.957 |
| rs4942556 | 13:47174585 | no | no | no | no | yes (10) | yes | no | yes | yes | yes | yes | no | yes | no | yes | 0.957 |
| rs1886964 | 13:47175084 | yes (1) | no | no | no | yes (8) | yes | yes | yes | yes | yes | yes | yes | yes | yes | yes | 0.957 |
| rs912428 | 13:47167903 | no | no | no | no | yes (3) | yes | no | yes | no | yes | yes | yes | yes | no | yes | 0.936 |
| rs7330453 | 13:47171755 | no | no | no | no | yes (1) | no | no | yes | no | yes | yes | no | no | no | yes | 0.936 |
| rs9534434 | 13:47172726 | no | no | no | no | yes (2) | no | no | no | no | no | yes | no | no | no | yes | 0.936 |
| rs6561322 | 13:47163185 | no | no | no | no | yes (2) | no | no | no | no | yes | yes | no | yes | no | yes | 0.930 |
| rs1570622 | 13:47169092 | no | no | no | no | yes (2) | no | no | yes | no | yes | yes | yes | yes | yes | yes | 0.930 |
| rs912427 | 13:47169273 | yes (1) | no | no | no | yes (4) | no | yes | yes | yes | yes | yes | yes | no | yes | yes | 0.930 |
| rs912426 | 13:47169419 | yes (1) | no | no | no | yes (4) | no | yes | yes | yes | yes | yes | yes | no | yes | yes | 0.930 |
| rs9567707 | 13:47175435 | yes (1) | no | no | no | yes (9) | yes | yes | yes | yes | yes | yes | yes | yes | yes | yes | 0.930 |
| rs912434 | 13:47189928 | no | no | yes (3) | no | yes (14) | yes | no | yes | yes | yes | yes | yes | yes | no | yes | 0.747 |
| rs4942557 | 13:47183068 | no | no | no | no | yes (14) | yes | no | yes | no | yes | yes | yes | no | no | no | 0.740 |
| rs6561323 | 13:47185601 | no | no | no | no | yes (3) | no | no | yes | no | no | yes | no | no | no | no | 0.740 |
| rs7335684 | 13:47193696 | no | no | no | no | yes (3) | no | yes | no | no | yes | yes | yes | no | no | yes | 0.738 |
| rs2181786 | 13:47199611 | no | no | no | no | yes (2) | no | no | no | no | yes | yes | no | no | no | yes | 0.729 |
| rs7318686 | 13:47200504 | no | no | no | no | yes (3) | no | no | no | no | no | yes | no | no | no | no | 0.729 |
| rs9526207 | 13:47201226 | no | no | no | no | yes (1) | no | no | no | no | no | yes | no | no | no | no | 0.729 |
| rs1570620 | 13:47201874 | no | no | no | no | yes (1) | no | no | no | no | yes | yes | no | no | no | yes | 0.729 |
| rs7986104 | 13:47206196 | no | no | no | no | yes (2) | no | no | no | yes | yes | yes | no | no | no | yes | 0.729 |
| rs4942558 | 13:47206582 | no | no | no | no | yes (2) | no | no | no | no | yes | yes | no | no | no | yes | 0.729 |
| rs4942559 | 13:47206814 | no | no | no | no | yes (2) | no | no | no | yes | yes | no | no | no | no | yes | 0.729 |
| rs9526208 | 13:47208767 | no | no | no | no | yes (3) | no | no | no | yes | no | yes | no | no | yes | yes | 0.729 |
| rs7318773 | 13:47208947 | no | no | no | no | yes (2) | no | no | no | no | no | yes | no | no | no | yes | 0.729 |
| rs1886220 | 13:47159484 | no | no | no | no | yes (2) | no | no | no | yes | no | yes | no | no | no | yes | 0.726 |
| rs1886219 | 13:47159881 | no | no | no | no | yes (1) | no | yes | yes | yes | yes | yes | no | no | no | yes | 0.726 |
| rs1535792 | 13:47165040 | no | no | no | no | yes (1) | no | no | yes | no | yes | yes | no | no | no | yes | 0.726 |
| rs7337752 | 13:47156855 | no | no | yes (3) | no | yes (9) | no | no | yes | no | yes | yes | no | no | no | yes | 0.725 |
| rs1535791 | 13:47165458 | no | no | no | no | yes (1) | no | no | yes | no | no | yes | no | no | no | yes | 0.713 |
| rs9316214 | 13:47157872 | no | yes (1) | yes (4) | no | yes (8) | no | no | yes | no | yes | yes | no | yes | no | yes | 0.712 |
| rs9534432 | 13:47157969 | no | yes (1) | yes (4) | no | yes (8) | no | no | yes | no | yes | yes | no | yes | no | yes | 0.712 |
| rs9534433 | 13:47158041 | no | yes (1) | yes (4) | no | yes (8) | no | no | yes | no | yes | yes | no | yes | no | yes | 0.712 |
| rs7328294 | 13:47159296 | no | no | no | no | yes (2) | no | no | no | yes | yes | yes | no | no | no | yes | 0.712 |
| rs6561321 | 13:47161560 | no | no | no | no | yes (4) | yes | yes | yes | yes | yes | yes | yes | yes | yes | yes | 0.712 |
| rs1123028 | 13:47166195 | no | no | no | no | yes (3) | yes | no | yes | no | yes | yes | yes | no | no | yes | 0.712 |
| rs1570621 | 13:47170118 | no | no | no | no | yes (5) | no | yes | yes | yes | yes | yes | no | no | yes | yes | 0.695 |
| rs8000003 | 13:47202798 | no | no | no | no | yes (1) | no | no | no | no | yes | yes | no | no | no | yes | 0.676 |
| rs1467604 | 13:47211879 | no | no | no | no | yes (1) | no | no | yes | no | yes | yes | no | no | no | yes | 0.673 |
| rs9316217 | 13:47209806 | no | no | no | no | yes (1) | no | no | no | no | no | no | no | no | no | no | 0.669 |
| rs1467605 | 13:47211861 | no | no | no | no | yes (1) | no | no | yes | no | yes | yes | no | no | no | yes | 0.669 |
| rs9534447 | 13:47212722 | no | no | no | no | yes (1) | no | no | yes | no | yes | no | yes | no | no | yes | 0.669 |
| rs9534448 | 13:47212961 | no | no | no | no | yes (1) | no | no | yes | no | yes | yes | yes | no | no | yes | 0.669 |
| rs9316222 | 13:47214690 | no | no | no | no | yes (1) | no | no | yes | yes | yes | yes | yes | no | no | yes | 0.669 |
| rs1408223 | 13:47215674 | no | no | no | no | yes (1) | yes | yes | yes | yes | yes | yes | yes | yes | yes | yes | 0.669 |
| rs4942566 | 13:47216011 | no | no | no | no | yes (1) | no | yes | yes | yes | yes | yes | yes | yes | yes | yes | 0.669 |
| rs1773118 | 13:47221640 | no | no | no | no | yes (2) | no | no | no | no | yes | yes | no | yes | no | no | 0.669 |
| rs1773122 | 13:47222433 | yes (1) | yes (2) | no | no | yes (2) | yes | no | yes | yes | yes | yes | no | yes | yes | yes | 0.669 |
| rs1773124 | 13:47223835 | no | no | no | no | yes (2) | no | no | yes | no | yes | yes | no | yes | yes | yes | 0.669 |
| rs9526212 | 13:47225745 | no | no | no | no | yes (1) | no | no | no | no | no | yes | no | yes | no | no | 0.669 |
| rs842406 | 13:47230194 | yes (1) | no | yes (3) | no | yes (17) | yes | yes | yes | yes | yes | yes | no | yes | yes | yes | 0.660 |
| rs1216862 | 13:47232678 | no | no | no | no | yes (5) | yes | no | no | no | yes | yes | yes | yes | yes | yes | 0.660 |
| rs842407 | 13:47228730 | no | no | no | no | yes (7) | no | no | yes | no | yes | yes | no | yes | no | no | 0.656 |
| rs1535793 | 13:47154966 | no | no | no | no | yes (1) | no | no | yes | no | no | yes | no | yes | yes | yes | 0.655 |
| rs4942553 | 13:47155975 | no | no | no | no | yes (1) | no | no | no | no | no | yes | yes | no | no | yes | 0.655 |
| rs4942561 | 13:47209347 | no | no | no | no | yes (2) | no | no | yes | no | yes | yes | no | no | no | yes | 0.655 |
| rs1747223 | 13:47224681 | no | no | no | no | yes (1) | no | no | no | no | no | yes | no | no | no | yes | 0.652 |
| rs842370 | 13:47238854 | no | no | yes (1) | no | yes (19) | no | no | yes | no | yes | yes | yes | yes | yes | yes | 0.631 |
| rs9534454 | 13:47234659 | yes (1) | no | yes (4) | no | yes (19) | no | no | no | no | yes | yes | yes | yes | no | yes | 0.623 |
| rs9526214 | 13:47237213 | no | no | yes (4) | no | yes (16) | no | no | yes | no | yes | yes | yes | yes | no | yes | 0.623 |
| rs7333028 | 13:47238684 | no | no | yes (2) | no | yes (19) | no | no | yes | no | yes | yes | yes | yes | no | yes | 0.623 |
| rs7319949 | 13:47148843 | no | no | no | no | yes (1) | no | no | yes | no | no | yes | no | no | no | no | 0.559 |
| rs7994173 | 13:47107188 | no | no | no | no | no | no | no | no | no | no | no | no | no | no | no | 0.528 |
| rs1570974 | 13:47108253 | no | no | no | no | no | no | no | no | no | no | no | no | no | no | no | 0.528 |
| rs1007171 | 13:47106226 | no | no | no | no | yes (2) | no | yes | no | no | no | yes | no | no | no | no | 0.526 |
| rs1326112 | 13:47100498 | yes (1) | no | no | no | yes (1) | yes | no | no | no | yes | yes | no | no | yes | yes | 0.523 |
| rs61948157 | 13:47095213 | no | no | no | no | no | no | no | no | yes | yes | yes | no | no | yes | yes | 0.522 |
| rs4942545 | 13:47095594 | no | no | no | no | no | yes | no | no | no | no | yes | yes | yes | yes | yes | 0.522 |
| rs7139769 | 13:47095886 | no | no | no | no | no | yes | no | no | no | no | yes | yes | yes | yes | yes | 0.522 |
| rs7317158 | 13:47096173 | no | no | no | no | no | yes | no | no | no | no | yes | yes | yes | yes | yes | 0.522 |
| rs9534416 | 13:47096271 | no | no | no | no | no | yes | no | no | no | yes | yes | yes | yes | yes | yes | 0.522 |
| rs9526188 | 13:47096658 | no | no | no | no | no | yes | no | yes | no | yes | yes | yes | yes | yes | yes | 0.522 |
| rs9526189 | 13:47097111 | no | no | no | no | no | no | no | yes | no | yes | yes | yes | yes | yes | yes | 0.522 |
| rs4942546 | 13:47097226 | no | no | no | no | no | yes | no | yes | no | yes | yes | yes | yes | yes | yes | 0.522 |
| rs4942547 | 13:47097227 | no | no | no | no | no | yes | no | yes | no | yes | yes | yes | yes | yes | yes | 0.522 |
| rs4942548 | 13:47097278 | no | no | no | no | no | yes | no | yes | no | yes | yes | yes | yes | yes | yes | 0.522 |
| rs7328711 | 13:47097643 | no | no | no | no | no | no | no | no | no | yes | yes | no | no | no | yes | 0.522 |
| rs7330387 | 13:47097802 | no | no | no | no | no | no | no | no | yes | yes | yes | no | no | yes | yes | 0.522 |
| rs1886967 | 13:47098648 | no | no | no | no | no | yes | yes | yes | yes | yes | yes | no | no | yes | yes | 0.522 |
| rs6561312 | 13:47098806 | no | no | no | no | no | no | no | yes | yes | yes | no | no | no | no | yes | 0.522 |
| rs6561313 | 13:47098897 | no | no | no | no | no | no | no | yes | yes | no | no | no | no | no | yes | 0.522 |
| rs6561314 | 13:47099042 | no | no | no | no | no | no | no | no | yes | yes | yes | no | no | no | yes | 0.522 |
| rs6561315 | 13:47099426 | no | no | no | no | no | no | no | no | no | yes | no | no | no | no | no | 0.522 |
| rs6561316 | 13:47099561 | no | no | no | no | no | no | no | no | no | yes | no | no | no | no | no | 0.522 |
| rs4942549 | 13:47099664 | no | no | no | no | no | no | no | no | no | yes | no | no | no | no | no | 0.522 |
| rs1326111 | 13:47100405 | yes (1) | no | no | no | yes (1) | yes | no | no | no | yes | yes | no | no | yes | yes | 0.522 |
| rs1326113 | 13:47100585 | no | no | no | no | yes (1) | yes | no | no | no | yes | yes | no | no | yes | yes | 0.522 |
| rs1570975 | 13:47103489 | no | no | no | no | yes (1) | no | no | no | no | no | yes | no | no | no | no | 0.522 |
| rs4942550 | 13:47104680 | no | no | no | no | yes (1) | no | no | yes | yes | yes | yes | no | no | no | no | 0.522 |
| rs9534425 | 13:47126767 | no | yes (2) | yes (3) | no | yes (27) | yes | yes | yes | yes | yes | yes | yes | yes | yes | yes | 0.521 |
| rs2405413 | 13:47113763 | no | no | no | no | no | no | no | no | no | no | no | no | no | no | no | 0.521 |
| rs7994252 | 13:47115608 | no | no | no | no | no | no | no | no | no | no | no | no | no | no | no | 0.521 |
| rs4941561 | 13:47118651 | no | no | no | no | no | no | no | no | no | no | yes | no | no | no | no | 0.521 |
| rs1012628 | 13:47119642 | no | no | no | no | no | no | no | no | no | no | no | no | no | no | no | 0.521 |
| rs4941563 | 13:47121510 | no | no | no | no | no | no | no | no | no | no | no | no | no | no | no | 0.521 |
| rs1886966 | 13:47094949 | no | no | no | no | no | no | no | no | yes | yes | yes | no | no | yes | yes | 0.517 |
| rs35441059 | 13:47095150 | no | no | no | no | no | no | no | no | yes | yes | yes | no | no | yes | yes | 0.517 |
| rs568025 | 13:47074083 | no | no | no | no | no | no | no | yes | no | no | no | no | no | no | no | 0.510 |
| rs493582 | 13:47075711 | no | no | no | no | no | no | no | no | no | no | no | no | no | no | no | 0.510 |
| rs642732 | 13:47077811 | no | no | no | no | no | no | no | no | no | no | no | no | no | no | no | 0.510 |
| rs643264 | 13:47078000 | no | no | no | no | no | no | no | no | no | no | no | no | no | no | no | 0.510 |
| rs71432911 | 13:47079657 | no | no | no | no | no | no | no | no | no | no | yes | no | no | no | no | 0.510 |
| rs9316216 | 13:47193442 | no | no | no | no | yes (3) | yes | no | no | no | yes | yes | yes | no | no | yes | 0.508 |
| rs7996004 | 13:47093502 | no | no | no | no | no | yes | no | no | yes | yes | yes | yes | no | yes | yes | 0.508 |
| rs13378860 | 13:47202652 | no | no | no | no | yes (1) | no | no | no | no | yes | yes | no | no | no | yes | 0.502 |

| **Supplementary Table 8**. Support vector machine learning model in-silico mutagenesis scores to predict functional effects of linked variants within the rs3802177 and *rs9526201* region (500 kb window, *R*^2^ thresholded at 0.20). | | | | | | | | | | | |
| --- | --- | --- | --- | --- | --- | --- | --- | --- | --- | --- | --- |
| Chr | RSID | Position | Reference allele | Alternative allele | Tag | MAX absolute delta | COLO205 | Healthy | Tumor | HCT116 | SW480 |
|  |  |  |  |  |  |  | (Ref[/]Alt) | (Ref[/]Alt) | (Ref[/]Alt) | (Ref[/]Alt) | (Ref[/]Alt) |
| rs3802177 region | | | | | | | | | | | |
| 8 | rs11558471 | 118185733 | G | A | rs3802177 | 0.593 | -0.292[/]-0.616 | -1.005[/]-1.388 | -0.374[/]-0.812 | -0.764[/]-1.234 | -0.228[[/]]-0.821 |
| 8 | rs28529793 | 118231951 | T | C | rs3802177 | 0.549 | -1.128[/]-1.033 | -0.825[/]-0.889 | -1.015[/]-0.465 | -1.14[/]-0.908 | -1.505[/]-1.131 |
| 8 | rs185166635 | 118209284 | A | C | rs3802177 | 0.48 | -2.353[/]-2.495 | -1.742[/]-1.646 | -2.083[/]-2.134 | -1.703[/]-1.938 | -1.402[/]-1.882 |
| 8 | rs4876711 | 118232337 | A | C | rs3802177 | 0.478 | -0.636[/]-0.483 | -0.075[/]0.175 | 0.122[/]0.601 | -0.765[/]-0.802 | -0.212[/]-0.093 |
| 8 | rs12549811 | 118232111 | T | G | rs3802177 | 0.456 | -0.898[/]-0.687 | -1.221[/]-0.898 | -0.536[/]-0.516 | -0.852[/]-0.711 | -1.044[/]-0.588 |
| 8 | rs13267156 | 118223515 | C | T | rs3802177 | 0.429 | -1.286[/]-1.37 | -2.082[/]-1.961 | -2.323[/]-1.894 | -1.854[/]-1.797 | -1.663[/]-1.428 |
| 8 | rs28552442 | 118231970 | T | C | rs3802177 | 0.392 | -1.106[/]-0.781 | -1.069[/]-0.676 | -0.586[/]-0.196 | -0.932[/]-0.591 | -1.048[/]-0.833 |
| 8 | rs3802177 | 118185025 | A | G | rs3802177 | 0.372 | -1.152[/]-0.985 | -1.72[/]-1.486 | -1.644[/]-1.389 | -1.329[/]-0.956 | -1.546[/]-1.228 |
| 8 | rs2466318 | 118217307 | T | C | rs3802177 | 0.363 | -1.741[/]-1.62 | -0.919[/]-1.282 | -1.23[/]-0.992 | -1.343[/]-1.202 | -1.609[/]-1.567 |
| 8 | rs2466293 | 118185938 | G | A | rs3802177 | 0.361 | -0.211[/]0.083 | -1.566[/]-1.267 | -1.4[/]-1.385 | -0.898[/]-0.699 | -1.056[/]-0.695 |
| 8 | rs4876710 | 118225130 | G | A | rs3802177 | 0.334 | -2.412[/]-2.471 | -1.845[/]-2.179 | -2.521[/]-2.71 | -2.048[/]-2.098 | -1.569[/]-1.846 |
| 8 | rs2466294 | 118185063 | G | C | rs3802177 | 0.321 | -1.013[/]-1.052 | -1.56[/]-1.637 | -1.314[/]-1.454 | -1.029[/]-1.221 | -1.062[/]-1.383 |
| 8 | rs13266634 | 118184783 | T | C | rs3802177 | 0.319 | 0.655[/]0.375 | -0.359[/]-0.455 | -0.549[/]-0.854 | -0.358[/]-0.039 | -0.697[/]-0.72 |
| 8 | rs7005548 | 118234187 | C | G | rs3802177 | 0.303 | -1.22[/]-1.319 | -1.376[/]-1.679 | -1.075[/]-1.155 | -1.492[/]-1.591 | -1.371[/]-1.423 |
| 8 | rs2466291 | 118190393 | G | A | rs3802177 | 0.285 | -1.46[/]-1.632 | -1.812[/]-1.909 | -1.866[/]-2.138 | -1.576[/]-1.686 | -1.264[/]-1.549 |
| 8 | rs1024069 | 118231280 | G | A | rs3802177 | 0.279 | -1.781[/]-2.06 | -1.902[/]-1.949 | -1.336[/]-1.442 | -1.031[/]-1.16 | -1.534[/]-1.617 |
| 8 | rs2464573 | 118222398 | G | A | rs3802177 | 0.27 | -0.214[/]-0.288 | -1.357[/]-1.209 | -1.136[/]-0.944 | -0.489[/]-0.219 | -0.752[/]-0.69 |
| 8 | rs2466292 | 118189531 | C | T | rs3802177 | 0.266 | -0.55[/]-0.816 | -0.852[/]-1.005 | -1.065[/]-1.062 | -1.482[/]-1.569 | -1.151[/]-1.326 |
| 8 | rs11774700 | 118220270 | C | T | rs3802177 | 0.263 | -0.663[/]-0.926 | -1.276[/]-1.388 | -0.841[/]-0.927 | -0.811[/]-0.95 | -0.671[/]-0.785 |
| 8 | rs12544930 | 118232242 | G | A | rs3802177 | 0.258 | -0.861[/]-0.95 | -0.258[/]-0.284 | 0.065[/]0.066 | -0.986[/]-0.756 | -0.815[/]-0.557 |
| 8 | rs2466295 | 118185041 | C | T | rs3802177 | 0.248 | -0.985[/]-1.233 | -1.586[/]-1.513 | -1.338[/]-1.435 | -1.033[/]-1.16 | -1.27[/]-1.251 |
| 8 | rs4300038 | 118217915 | A | G | rs3802177 | 0.247 | -2.811[/]-2.818 | -2.625[/]-2.508 | -2.184[/]-2.431 | -2.13[/]-2.225 | -2.757[/]-2.78 |
| 8 | rs2466288 | 118200188 | G | C | rs3802177 | 0.224 | -2.433[/]-2.47 | -1.87[/]-1.646 | -1.789[/]-1.874 | -1.744[/]-1.946 | -1.902[/]-1.853 |
| 8 | rs4350011 | 118225198 | T | G | rs3802177 | 0.222 | -2.797[/]-2.745 | -1.948[/]-2.17 | -2.455[/]-2.598 | -2.11[/]-2.175 | -1.662[/]-1.639 |
| 8 | rs1995222 | 118229762 | T | C | rs3802177 | 0.2 | -1.153[/]-1.116 | -1.074[/]-1.142 | -1.069[/]-1.105 | -1.813[/]-1.778 | -1.944[/]-1.744 |
| 8 | rs1024068 | 118231642 | C | G | rs3802177 | 0.196 | -0.637[/]-0.572 | -0.598[/]-0.733 | -0.452[/]-0.256 | -0.133[/]-0.079 | -0.865[/]-0.876 |
| 8 | rs2466311 | 118221096 | G | C | rs3802177 | 0.193 | -1.126[/]-1.281 | -1.406[/]-1.494 | -1.035[/]-1.227 | -1.474[/]-1.569 | -1.4[/]-1.416 |
| 8 | rs997314 | 118236971 | T | C | rs3802177 | 0.173 | -0.133[/]-0.165 | 0.107[/]-0.066 | -0.054[/]-0.022 | 0.276[/]0.43 | -0.232[/]-0.141 |
| 8 | rs12545397 | 118236191 | C | T | rs3802177 | 0.171 | -1.23[/]-1.067 | -1.724[/]-1.68 | -0.461[/]-0.375 | -1.035[/]-0.864 | -0.81[/]-0.784 |
| 8 | rs35859536 | 118191475 | T | C | rs3802177 | 0.168 | -0.384[/]-0.216 | -0.28[/]-0.296 | 0.071[/]0.079 | -0.129[/]0.034 | -0.726[/]-0.75 |
| 8 | rs9650069 | 118204020 | T | C | rs3802177 | 0.167 | -1.129[/]-0.963 | -1.7[/]-1.685 | -1.633[/]-1.616 | -1.267[/]-1.252 | -1.339[/]-1.348 |
| 8 | rs2466320 | 118216673 | G | T | rs3802177 | 0.164 | -0.818[/]-0.911 | -0.677[/]-0.84 | -1.242[/]-1.292 | -0.781[/]-0.873 | -0.906[/]-0.931 |
| 8 | rs951337 | 118230236 | T | C | rs3802177 | 0.164 | -2.282[/]-2.372 | -1.306[/]-1.142 | -1.598[/]-1.755 | -1.804[/]-1.861 | -1.624[/]-1.549 |
| 8 | rs112129735 | 118224482 | T | C | rs3802177 | 0.153 | -1.543[/]-1.39 | -1.298[/]-1.295 | -1.354[/]-1.418 | -1.76[/]-1.877 | -2.068[/]-2.042 |
| 8 | rs7005140 | 118234147 | G | A | rs3802177 | 0.15 | -0.984[/]-1.034 | -1.211[/]-1.112 | -0.473[/]-0.623 | -1.034[/]-1.113 | -0.925[/]-0.858 |
| 8 | rs10955807 | 118236571 | A | G | rs3802177 | 0.147 | -2.029[/]-1.984 | -1.179[/]-1.326 | -1.343[/]-1.315 | -2.039[/]-2.047 | -1.526[/]-1.48 |
| 8 | rs10584444 | 118194484 | TTAAAA | T | rs3802177 | 0.121 | -1.688[/]-1.629 | -2.02[/]-2.048 | -2.005[/]-1.961 | -2.144[/]-2.1 | -1.764[/]-1.644 |
| 8 | rs12545006 | 118232629 | G | A | rs3802177 | 0.117 | -1.175[/]-1.22 | -1.155[/]-1.272 | -1.702[/]-1.816 | -1.256[/]-1.222 | -1.272[/]-1.332 |
| 8 | rs997313 | 118236975 | T | A | rs3802177 | 0.107 | -0.191[/]-0.12 | -0.178[/]-0.078 | -0.086[/]-0.029 | 0.351[/]0.458 | -0.205[/]-0.126 |
| 8 | rs538658564 | 118225701 | G | GAACTAACT | rs3802177 | 0.098 | -1.386[/]-1.365 | -2.848[/]-2.884 | -2.196[/]-2.262 | -1.901[/]-1.963 | -2.018[/]-2.116 |
| 8 | rs2466289 | 118195570 | G | A | rs3802177 | 0.081 | -1.477[/]-1.445 | -1.272[/]-1.353 | -1.143[/]-1.185 | -1.512[/]-1.469 | -1.71[/]-1.771 |
| 8 | rs56951566 | 118216746 | G | GA | rs3802177 | 0.064 | -1.409[/]-1.377 | -0.683[/]-0.63 | -1.226[/]-1.162 | -0.945[/]-0.915 | -0.791[/]-0.754 |
| rs9526201 region | | | | | | | | | | | |
| 13 | rs9534444 | 47204385 | C | G | rs9526201 | 1.148 | -0.487[/]-0.902 | -0.335[/]-1.064 | -0.465[/]-1.296 | -0.217[/]-1.364 | -0.128[/]-1.033 |
| 13 | rs1142184 | 47187453 | A | G | rs9526201 | 0.981 | -2.05[/]-2.602 | -1.678[/]-2.325 | -1.284[/]-2.265 | -1.298[/]-1.947 | -1.014[/]-1.777 |
| 13 | rs7983898 | 47245633 | G | C | rs9526201 | 0.953 | -1.006[/]-1.734 | -1.085[/]-2.038 | -1.06[/]-1.848 | -1.492[/]-2.037 | -1.649[/]-2.3 |
| 13 | rs9316222 | 47214690 | T | C | rs9526201 | 0.834 | -1.441[/]-1.857 | -1.934[/]-2.464 | -1.662[/]-2.217 | -1.046[/]-1.457 | -1.624[/]-2.458 |
| 13 | rs56058552 | 47244958 | T | TCAGGGACAGGGACAGGGA | rs9526201 | 0.792 | -1.157[/]-1.384 | -1.142[/]-1.563 | -1.02[/]-1.812 | -1.527[/]-1.912 | -1.779[/]-2.356 |
| 13 | rs4941564 | 47242761 | A | G | rs9526201 | 0.79 | 0.718[/]0.562 | 0.705[/]-0.084 | 0.41[/]-0.287 | 0.617[/]-0.109 | 0.314[/]-0.097 |
| 13 | rs7986467 | 47241223 | C | T | rs9526201 | 0.764 | -0.921[/]-1.1 | -0.459[/]-0.86 | -0.75[/]-1.162 | -0.014[/]-0.577 | -0.593[/]-1.357 |
| 13 | rs1886222 | 47243476 | G | C | rs9526201 | 0.756 | -0.118[/]0.199 | 1.005[/]1.563 | 0.548[/]1.305 | -0.153[/]0.099 | 0.254[/]0.637 |
| 13 | rs9526209 | 47219780 | T | C | rs9526201 | 0.741 | -2.075[/]-1.335 | -1.909[/]-1.466 | -2.116[/]-1.543 | -1.052[/]-0.477 | -1.474[/]-0.788 |
| 13 | rs1408224 | 47215218 | G | A | rs9526201 | 0.722 | 0.059[/]0.753 | 0.308[/]1.03 | 0.199[/]0.459 | 0.642[/]1.1 | -0.058[/]0.466 |
| 13 | rs12864576 | 47211620 | C | T | rs9526201 | 0.68 | -0.388[/]-0.099 | -0.651[/]0.03 | -0.425[/]-0.069 | -0.067[/]0.279 | -0.732[/]-0.466 |
| 13 | rs1773122 | 47222433 | A | G | rs9526201 | 0.675 | 0.698[/]0.971 | 0.355[/]0.857 | 0.159[/]0.834 | 0.655[/]0.797 | 0.228[/]0.671 |
| 13 | rs1216859 | 47231911 | G | A | rs9526201 | 0.674 | -1.501[/]-2.016 | -1.735[/]-2.409 | -2.114[/]-2.306 | -2.085[/]-2.388 | -1.899[/]-2.207 |
| 13 | rs7983902 | 47245644 | G | C | rs9526201 | 0.658 | -1.028[/]-0.599 | -1.181[/]-0.833 | -1.073[/]-0.812 | -1.441[/]-0.783 | -1.623[/]-1.228 |
| 13 | rs9534458 | 47237952 | G | A | rs9526201 | 0.656 | 0.116[/]0.203 | -0.242[/]0.413 | 0.365[/]0.952 | -0.365[/]-0.133 | -0.008[/]0.025 |
| 13 | rs1408223 | 47215674 | A | G | rs9526201 | 0.653 | -0.488[/]-0.568 | -0.378[/]-0.697 | 0.407[/]-0.053 | -0.754[/]-1.23 | -0.105[/]-0.758 |
| 13 | rs1326111 | 47100405 | T | C | rs9526201 | 0.624 | 0.631[/]0.234 | 0.117[/]0.003 | 0.354[/]0.125 | -0.441[/]-1.064 | -0.141[/]-0.611 |
| 13 | rs4942556 | 47174585 | C | T | rs9526201 | 0.599 | -0.888[/]-1.199 | 1.03[/]0.437 | 0.511[/]-0.088 | -1.066[/]-1.272 | -0.613[/]-1.124 |
| 13 | rs927624 | 47270040 | T | A | rs9526201 | 0.594 | -1.166[/]-1.039 | 0.226[/]-0.367 | -0.934[/]-1.521 | -1.639[/]-1.613 | -1.885[/]-2.002 |
| 13 | rs7985741 | 47191647 | A | C | rs9526201 | 0.587 | 0.094[/]0.318 | -0.543[/]-0.447 | -0.468[/]-0.322 | -0.338[/]-0.285 | -0.932[/]-0.345 |
| 13 | rs4942546 | 47097226 | A | G | rs9526201 | 0.585 | -1.14[/]-0.708 | -1.077[/]-0.559 | -1.45[/]-1.247 | -1.536[/]-1.254 | -1.56[/]-0.975 |
| 13 | rs842370 | 47238854 | A | G | rs9526201 | 0.585 | -0.095[/]-0.073 | 0.954[/]1.539 | -0.254[/]0.157 | -0.921[/]-0.891 | -0.315[/]-0.058 |
| 13 | rs1216860 | 47232268 | C | T | rs9526201 | 0.572 | -1.143[/]-1.715 | 0.232[/]-0.253 | -0.876[/]-1.325 | -1.189[/]-1.543 | -0.517[/]-0.706 |
| 13 | rs842393 | 47277029 | C | A | rs9526201 | 0.538 | 0.213[/]-0.241 | -0.653[/]-1.191 | -0.511[/]-0.888 | -0.124[/]-0.656 | -0.629[/]-1.118 |
| 13 | rs7318773 | 47208947 | G | T | rs9526201 | 0.533 | 1.198[/]1.376 | 0.147[/]0.205 | 0.871[/]0.863 | 1.322[/]0.79 | 1.102[/]0.745 |
| 13 | rs35441059 | 47095150 | T | C | rs9526201 | 0.527 | -2.5[/]-2.29 | -2.717[/]-2.752 | -2.827[/]-2.576 | -2.401[/]-1.951 | -3.345[/]-2.817 |
| 13 | rs2181786 | 47199611 | T | C | rs9526201 | 0.525 | -2.072[/]-1.863 | -2.53[/]-2.454 | -2.702[/]-2.884 | -2.658[/]-2.133 | -1.608[/]-1.717 |
| 13 | rs11421583 | 47174295 | T | TA | rs9526201 | 0.521 | -1.627[/]-1.106 | -1.07[/]-0.833 | -1.239[/]-1.112 | -1.088[/]-0.714 | -1.42[/]-1.308 |
| 13 | rs9534460 | 47240933 | T | G | rs9526201 | 0.518 | -0.868[/]-0.42 | -1.417[/]-0.899 | -1.525[/]-1.451 | -1.185[/]-0.912 | -1.482[/]-1.319 |
| 13 | rs4942568 | 47236989 | A | G | rs9526201 | 0.494 | -0.372[/]0.029 | -0.745[/]-0.587 | -0.926[/]-0.566 | -1.008[/]-0.621 | -0.761[/]-0.268 |
| 13 | rs6561327 | 47296903 | C | T | rs9526201 | 0.494 | -0.294[/]-0.105 | -0.458[/]-0.075 | -0.138[/]0.356 | -0.08[/]-0.238 | -0.565[/]-0.304 |
| 13 | rs9567688 | 47085718 | A | G | rs9526201 | 0.49 | -1.421[/]-1.098 | -1.814[/]-1.325 | -1.894[/]-1.666 | -1.556[/]-1.464 | -1.71[/]-1.585 |
| 13 | rs9534463 | 47246824 | C | G | rs9526201 | 0.488 | -1.089[/]-0.601 | -0.828[/]-0.653 | -1.358[/]-0.939 | -1.018[/]-0.896 | -1.171[/]-0.878 |
| 13 | rs842378 | 47260841 | A | G | rs9526201 | 0.487 | -1.583[/]-1.378 | -1.062[/]-0.912 | -0.888[/]-0.434 | -1.417[/]-0.931 | -1.775[/]-1.34 |
| 13 | rs9534432 | 47157969 | C | T | rs9526201 | 0.467 | -1.072[/]-0.855 | -1.657[/]-1.24 | -1.325[/]-0.912 | -0.882[/]-0.586 | -1.469[/]-1.002 |
| 13 | rs1536292 | 47083727 | C | T | rs9526201 | 0.462 | -0.492[/]-0.821 | -0.009[/]-0.361 | -0.571[/]-1.029 | 0.289[/]-0.173 | -0.293[/]-0.482 |
| 13 | rs7335684 | 47193696 | G | A | rs9526201 | 0.454 | -1.989[/]-1.987 | -1.519[/]-1.688 | -2.279[/]-2.217 | -2.275[/]-2.111 | -1.986[/]-1.532 |
| 13 | rs842394 | 47276504 | C | G | rs9526201 | 0.454 | -2.904[/]-3.061 | -2.649[/]-2.647 | -2.306[/]-2.76 | -2.374[/]-2.547 | -2.758[/]-2.681 |
| 13 | rs9534461 | 47241834 | T | A | rs9526201 | 0.454 | -2.682[/]-2.447 | -1.604[/]-1.231 | -1.967[/]-1.514 | -1.606[/]-1.526 | -2.07[/]-1.738 |
| 13 | rs4941558 | 47081830 | A | G | rs9526201 | 0.452 | -1.25[/]-0.797 | -1.771[/]-1.529 | -1.466[/]-1.361 | -1.419[/]-1.363 | -1.454[/]-1.082 |
| 13 | rs7318686 | 47200504 | G | A | rs9526201 | 0.451 | -0.94[/]-1.022 | -1.365[/]-1.584 | -1.272[/]-1.723 | -1.2[/]-1.248 | -1.088[/]-1.51 |
| 13 | rs9534459 | 47238950 | T | A | rs9526201 | 0.448 | -0.511[/]-0.471 | 0.954[/]0.506 | -0.003[/]-0.347 | -1.37[/]-1.247 | -0.842[/]-0.993 |
| 13 | rs4344620 | 47266990 | A | G | rs9526201 | 0.446 | -1.553[/]-1.528 | -1.185[/]-1.282 | -0.961[/]-1.407 | -1.42[/]-1.689 | -1.638[/]-1.703 |
| 13 | rs13378860 | 47202652 | C | T | rs9526201 | 0.444 | -1.303[/]-1.383 | -1.633[/]-1.189 | -1.379[/]-1.486 | -1.075[/]-1.022 | -1.376[/]-1.567 |
| 13 | rs912427 | 47169273 | T | C | rs9526201 | 0.438 | -1.82[/]-1.383 | -0.715[/]-0.739 | -1.283[/]-1.097 | -1.48[/]-1.407 | -1.136[/]-1.055 |
| 13 | rs9534453 | 47230657 | G | A | rs9526201 | 0.437 | -0.695[/]-0.954 | -1.322[/]-1.759 | -1.332[/]-1.689 | -0.894[/]-1.039 | -0.797[/]-0.946 |
| 13 | rs7330493 | 47156263 | C | T | rs9526201 | 0.431 | -1.736[/]-1.894 | -1.424[/]-1.73 | -1.351[/]-1.589 | -1.244[/]-1.328 | -0.982[/]-1.413 |
| 13 | rs643264 | 47078000 | C | T | rs9526201 | 0.424 | -1.711[/]-2.051 | -0.634[/]-1.059 | -1.296[/]-1.588 | -1.744[/]-1.732 | -2.009[/]-2.278 |
| 13 | rs4942564 | 47213980 | T | C | rs9526201 | 0.423 | -1.526[/]-1.104 | -0.965[/]-0.96 | -0.854[/]-0.961 | -1.356[/]-1.123 | -1.777[/]-1.623 |
| 13 | rs1886220 | 47159484 | A | G | rs9526201 | 0.41 | -2.276[/]-1.866 | -2.293[/]-1.966 | -2.164[/]-1.836 | -1.421[/]-1.263 | -1.621[/]-1.292 |
| 13 | rs4942548 | 47097278 | G | C | rs9526201 | 0.409 | -0.765[/]-0.433 | -0.961[/]-0.758 | -1.297[/]-0.998 | -1.79[/]-1.381 | -1.56[/]-1.341 |
| 13 | rs842399 | 47274478 | A | T | rs9526201 | 0.404 | 0.286[/]0.052 | -0.056[/]-0.217 | -0.509[/]-0.644 | 0.105[/]0.069 | -0.19[/]-0.594 |
| 13 | rs842401 | 47271006 | T | C | rs9526201 | 0.402 | -2.291[/]-2.693 | -2.847[/]-3.199 | -2.457[/]-2.646 | -1.662[/]-1.765 | -2.953[/]-2.916 |
| 13 | rs9567716 | 47241532 | T | C | rs9526201 | 0.397 | -1.067[/]-0.826 | -1.53[/]-1.397 | -1.072[/]-0.929 | -0.888[/]-0.751 | -1.178[/]-0.781 |
| 13 | rs1747227 | 47221039 | G | A | rs9526201 | 0.393 | -3.371[/]-3.492 | -3.113[/]-3.255 | -2.955[/]-2.873 | -2.664[/]-3.057 | -2.512[/]-2.61 |
| 13 | rs4942562 | 47210710 | G | A | rs9526201 | 0.393 | -0.423[/]-0.03 | -0.791[/]-0.634 | -1.328[/]-1.015 | -2.11[/]-2.007 | -1.613[/]-1.402 |
| 13 | rs11840358 | 47125701 | G | A | rs9526201 | 0.391 | 0.049[/]-0.342 | -0.175[/]-0.481 | -0.425[/]-0.605 | 0.342[/]0.032 | 0.443[/]0.189 |
| 13 | rs7328294 | 47159296 | T | A | rs9526201 | 0.39 | -1.028[/]-0.857 | -1.137[/]-1.527 | -1.112[/]-1.294 | -0.9[/]-0.826 | -0.615[/]-0.479 |
| 13 | rs34706092 | 47304608 | G | A | rs9526201 | 0.384 | -0.499[/]-0.5 | 0.218[/]0.184 | 0.461[/]0.077 | -0.24[/]-0.412 | -0.313[/]-0.509 |
| 13 | rs9590974 | 47238717 | C | A | rs9526201 | 0.383 | -0.398[/]-0.233 | 0.162[/]0.372 | -0.703[/]-0.32 | -0.448[/]-0.408 | -0.444[/]-0.212 |
| 13 | rs7328711 | 47097643 | C | T | rs9526201 | 0.38 | -2.243[/]-2.086 | -1.966[/]-1.899 | -2.034[/]-1.76 | -2.439[/]-2.059 | -1.947[/]-1.683 |
| 13 | rs7317158 | 47096173 | G | T | rs9526201 | 0.378 | -0.832[/]-0.972 | -0.065[/]-0.149 | -0.221[/]-0.216 | 0.19[/]-0.188 | -0.315[/]-0.418 |
| 13 | rs842395 | 47276470 | G | C | rs9526201 | 0.377 | -2.955[/]-3.14 | -2.615[/]-2.983 | -2.497[/]-2.875 | -2.496[/]-2.616 | -3.039[/]-3.209 |
| 13 | rs1969899 | 47083758 | T | C | rs9526201 | 0.375 | -0.53[/]-0.788 | -0.104[/]-0.247 | -0.669[/]-1.044 | -0.24[/]-0.184 | -0.596[/]-0.633 |
| 13 | rs1747221 | 47225573 | G | T | rs9526201 | 0.374 | -2.073[/]-2.218 | -1.656[/]-1.768 | -1.822[/]-2.196 | -0.597[/]-0.928 | -2.401[/]-2.446 |
| 13 | rs842397 | 47274727 | T | C | rs9526201 | 0.372 | -0.721[/]-1.093 | -0.845[/]-1.122 | -0.804[/]-1.019 | -1.028[/]-1.338 | -1.155[/]-1.422 |
| 13 | rs7986036 | 47240876 | G | A | rs9526201 | 0.37 | -0.818[/]-0.776 | -1.1[/]-1.149 | -1.296[/]-0.926 | -1.324[/]-1.28 | -0.94[/]-0.807 |
| 13 | rs4284534 | 47267033 | G | A | rs9526201 | 0.369 | -1.205[/]-1.253 | -0.947[/]-0.916 | -0.701[/]-1.071 | -0.661[/]-0.929 | -1.287[/]-1.512 |
| 13 | rs4942566 | 47216011 | G | C | rs9526201 | 0.366 | -0.161[/]0.206 | 0.113[/]0.442 | -0.308[/]-0.256 | -0.383[/]-0.482 | 0.171[/]0.381 |
| 13 | rs9534447 | 47212722 | G | C | rs9526201 | 0.366 | -0.659[/]-0.736 | -0.623[/]-0.775 | -0.509[/]-0.875 | -0.512[/]-0.669 | -0.892[/]-1.246 |
| 13 | rs9534454 | 47234659 | T | C | rs9526201 | 0.365 | -0.68[/]-0.412 | -0.585[/]-0.349 | -0.375[/]-0.154 | -0.454[/]-0.256 | -1.409[/]-1.044 |
| 13 | rs35014736 | 47219240 | AG | A | rs9526201 | 0.362 | -1.022[/]-1.211 | -0.162[/]-0.524 | -0.874[/]-0.99 | -1.209[/]-1.543 | -1.359[/]-1.564 |
| 13 | rs1216862 | 47232678 | A | G | rs9526201 | 0.361 | -1.277[/]-1.448 | -1.694[/]-2.025 | -1.665[/]-2.026 | -1.05[/]-1.363 | -0.681[/]-0.956 |
| 13 | rs9534425 | 47126767 | G | A | rs9526201 | 0.359 | -0.578[/]-0.937 | -1.518[/]-1.583 | -0.766[/]-0.792 | -1.034[/]-1.13 | -1.356[/]-1.456 |
| 13 | rs9316214 | 47157872 | C | T | rs9526201 | 0.355 | -0.772[/]-0.873 | -0.498[/]-0.844 | -0.696[/]-1.051 | -1.126[/]-1.155 | -0.677[/]-0.857 |
| 13 | rs1750498 | 47062093 | A | C | rs9526201 | 0.352 | -1.151[/]-0.799 | -1.622[/]-1.467 | -1.166[/]-1.016 | -1.228[/]-1.191 | -1.149[/]-0.928 |
| 13 | rs4245338 | 47244162 | G | A | rs9526201 | 0.35 | -0.566[/]-0.545 | -1.097[/]-1.149 | -0.627[/]-0.813 | -0.741[/]-1.091 | -0.507[/]-0.746 |
| 13 | rs1535792 | 47165040 | T | A | rs9526201 | 0.349 | -1.256[/]-0.921 | -0.763[/]-0.568 | -1.716[/]-1.597 | -1.58[/]-1.353 | -1.78[/]-1.43 |
| 13 | rs4942559 | 47206814 | G | C | rs9526201 | 0.345 | -1.43[/]-1.36 | -1.849[/]-1.663 | -1.668[/]-1.323 | -1.37[/]-1.29 | -0.827[/]-0.777 |
| 13 | rs842392 | 47277099 | A | T | rs9526201 | 0.345 | -0.488[/]-0.326 | -0.405[/]-0.06 | -0.293[/]-0.015 | -0.514[/]-0.486 | -0.811[/]-0.697 |
| 13 | rs4941560 | 47088292 | T | C | rs9526201 | 0.343 | -0.284[/]-0.487 | -1.179[/]-1.248 | -0.751[/]-0.877 | -0.336[/]-0.625 | 0.056[/]-0.287 |
| 13 | rs1216864 | 47233437 | C | T | rs9526201 | 0.339 | -2.879[/]-2.54 | -3.001[/]-2.726 | -2.637[/]-2.462 | -2.37[/]-2.086 | -2.492[/]-2.378 |
| 13 | rs9534439 | 47192049 | T | C | rs9526201 | 0.339 | -1.848[/]-1.917 | -0.809[/]-0.86 | -0.806[/]-0.985 | -0.76[/]-0.983 | -1.237[/]-1.575 |
| 13 | rs1123028 | 47166195 | C | T | rs9526201 | 0.338 | -0.514[/]-0.741 | -1.078[/]-1.251 | -1.388[/]-1.705 | -1.098[/]-1.345 | -1.012[/]-1.349 |
| 13 | rs11441375 | 47272621 | G | GA | rs9526201 | 0.338 | -0.706[/]-1.044 | -1.855[/]-1.879 | -1.355[/]-1.6 | -0.808[/]-0.755 | -0.656[/]-0.688 |
| 13 | rs1216863 | 47232741 | A | G | rs9526201 | 0.335 | -1.548[/]-1.438 | -2.087[/]-1.817 | -1.81[/]-1.514 | -1.391[/]-1.114 | -1.102[/]-0.767 |
| 13 | rs1773133 | 47221220 | G | C | rs9526201 | 0.335 | -2.45[/]-2.514 | -2.024[/]-2.359 | -1.679[/]-2.001 | -1.754[/]-1.881 | -1.343[/]-1.517 |
| 13 | rs4941561 | 47118651 | C | T | rs9526201 | 0.335 | -0.545[/]-0.763 | -0.794[/]-1.08 | -1.049[/]-1.185 | -1.155[/]-1.49 | -1.288[/]-1.475 |
| 13 | rs2147163 | 47269793 | T | C | rs9526201 | 0.334 | -0.876[/]-0.827 | -0.633[/]-0.496 | -0.905[/]-0.571 | -0.727[/]-0.57 | -0.993[/]-0.698 |
| 13 | rs9526201 | 47191972 | G | A | rs9526201 | 0.333 | -1.298[/]-1.592 | 0.02[/]-0.127 | -0.576[/]-0.665 | -0.298[/]-0.422 | -0.817[/]-1.15 |
| 13 | rs4942558 | 47206582 | T | C | rs9526201 | 0.332 | -1.579[/]-1.248 | -1.208[/]-1.046 | -1.139[/]-0.999 | -0.971[/]-0.746 | -0.967[/]-0.803 |
| 13 | rs7333782 | 47293969 | T | C | rs9526201 | 0.33 | -3.326[/]-3.485 | -2.552[/]-2.574 | -2.638[/]-2.835 | -2.358[/]-2.488 | -2.961[/]-3.291 |
| 13 | rs1773124 | 47223835 | G | A | rs9526201 | 0.328 | -1.677[/]-1.348 | -1.449[/]-1.513 | -1.709[/]-1.634 | -2.388[/]-2.29 | -1.804[/]-1.724 |
| 13 | rs9567718 | 47252742 | T | C | rs9526201 | 0.328 | -0.772[/]-0.892 | -0.708[/]-0.997 | -0.439[/]-0.469 | -0.798[/]-0.957 | -0.448[/]-0.776 |
| 13 | rs7139769 | 47095886 | G | A | rs9526201 | 0.324 | -0.253[/]-0.559 | -0.29[/]-0.613 | -0.322[/]-0.586 | 0.037[/]-0.272 | -0.376[/]-0.49 |
| 13 | rs1467604 | 47211879 | G | A | rs9526201 | 0.322 | -0.315[/]-0.483 | -0.297[/]-0.511 | -0.755[/]-0.731 | -0.258[/]-0.58 | -0.738[/]-0.726 |
| 13 | rs1747224 | 47223102 | G | A | rs9526201 | 0.322 | -1.865[/]-1.996 | -1.428[/]-1.578 | -1.581[/]-1.903 | -1.984[/]-2.068 | -1.797[/]-2.009 |
| 13 | rs1324007 | 47245318 | C | T | rs9526201 | 0.321 | -0.332[/]-0.276 | 0.074[/]-0.145 | -0.325[/]-0.463 | -0.427[/]-0.533 | -0.731[/]-1.052 |
| 13 | rs842389 | 47278494 | C | A | rs9526201 | 0.321 | -1.907[/]-1.866 | -1.347[/]-1.025 | -1.308[/]-1.24 | -1.361[/]-1.277 | -1.424[/]-1.194 |
| 13 | rs1886219 | 47159881 | A | T | rs9526201 | 0.32 | -0.811[/]-0.664 | -0.771[/]-0.72 | -0.888[/]-0.762 | -0.402[/]-0.722 | -0.251[/]-0.126 |
| 13 | rs35383971 | 47226158 | G | T | rs9526201 | 0.32 | -2.105[/]-1.895 | -2.158[/]-1.838 | -1.827[/]-1.686 | -0.963[/]-0.927 | -1.79[/]-1.79 |
| 13 | rs7994252 | 47115608 | A | G | rs9526201 | 0.32 | -0.467[/]-0.756 | -0.849[/]-1.1 | -0.057[/]-0.27 | -0.746[/]-0.774 | -0.758[/]-1.078 |
| 13 | rs842388 | 47278740 | T | G | rs9526201 | 0.32 | -2.621[/]-2.654 | -1.439[/]-1.529 | -1.873[/]-2.193 | -2.115[/]-2.072 | -1.818[/]-1.905 |
| 13 | rs7993645 | 47242088 | T | C | rs9526201 | 0.319 | -1.307[/]-1.028 | -1.613[/]-1.336 | -1.425[/]-1.176 | -2.081[/]-1.848 | -0.708[/]-0.389 |
| 13 | rs912426 | 47169419 | C | T | rs9526201 | 0.315 | -0.851[/]-1.076 | -0.065[/]-0.13 | -0.367[/]-0.481 | -0.677[/]-0.814 | -0.554[/]-0.869 |
| 13 | rs706621 | 47273458 | G | C | rs9526201 | 0.313 | -0.217[/]-0.164 | -0.056[/]-0.368 | -0.067[/]-0.135 | 0.338[/]0.448 | 0.197[/]0.19 |
| 13 | rs842405 | 47270592 | A | G | rs9526201 | 0.308 | -0.819[/]-0.698 | -1.19[/]-1.169 | -0.83[/]-0.692 | -1.03[/]-0.834 | -0.986[/]-0.678 |
| 13 | rs1886221 | 47243433 | G | A | rs9526201 | 0.307 | -0.135[/]-0.442 | 0.239[/]0.231 | 0.005[/]-0.162 | -0.43[/]-0.5 | -0.09[/]-0.33 |
| 13 | rs71432910 | 47070600 | T | A | rs9526201 | 0.302 | -2.094[/]-2.097 | -1.301[/]-1.437 | -1.568[/]-1.87 | -1.393[/]-1.505 | -1.743[/]-1.741 |
| 13 | rs7330389 | 47075444 | G | A | rs9526201 | 0.302 | -1.09[/]-1.294 | -1.542[/]-1.844 | -1.271[/]-1.453 | -0.591[/]-0.546 | -1.052[/]-1.224 |
| 13 | rs842382 | 47259619 | A | G | rs9526201 | 0.302 | -0.594[/]-0.557 | -0.499[/]-0.197 | -0.505[/]-0.316 | -0.277[/]-0.506 | -0.445[/]-0.272 |
| 13 | rs4942569 | 47243883 | C | T | rs9526201 | 0.301 | -0.833[/]-0.885 | -1.342[/]-1.225 | -1.265[/]-0.964 | -1.389[/]-1.216 | -0.88[/]-0.816 |
| 13 | rs842391 | 47277212 | T | C | rs9526201 | 0.299 | -0.028[/]0.189 | -0.348[/]-0.202 | 0.048[/]0.347 | -0.543[/]-0.433 | 0.126[/]0.241 |
| 13 | rs71432911 | 47079657 | G | A | rs9526201 | 0.297 | -0.741[/]-1.019 | -0.409[/]-0.6 | -0.849[/]-0.938 | -0.779[/]-0.922 | -1.014[/]-1.311 |
| 13 | rs7321115 | 47174334 | A | G | rs9526201 | 0.297 | -1.097[/]-1.074 | -0.461[/]-0.201 | -0.956[/]-0.733 | -1.111[/]-0.938 | -1.018[/]-0.721 |
| 13 | rs842376 | 47261390 | A | G | rs9526201 | 0.297 | -2.49[/]-2.394 | -1.79[/]-1.857 | -2.114[/]-1.968 | -1.518[/]-1.337 | -2.428[/]-2.131 |
| 13 | rs17068697 | 47293815 | G | A | rs9526201 | 0.294 | -1.776[/]-2.07 | -1.095[/]-1.362 | -1.582[/]-1.618 | -1.31[/]-1.486 | -1.834[/]-2.031 |
| 13 | rs842387 | 47279454 | A | G | rs9526201 | 0.293 | -0.161[/]-0.331 | -0.551[/]-0.83 | -0.521[/]-0.814 | -0.497[/]-0.748 | -0.327[/]-0.554 |
| 13 | rs912434 | 47189928 | G | T | rs9526201 | 0.293 | -1.058[/]-1.351 | -1.6[/]-1.689 | -1.449[/]-1.476 | -0.741[/]-0.82 | -1.23[/]-1.301 |
| 13 | rs113541848 | 47068146 | T | C | rs9526201 | 0.289 | -2.571[/]-2.392 | -2.282[/]-2.087 | -2.347[/]-2.346 | -1.818[/]-1.529 | -2.377[/]-2.211 |
| 13 | rs842375 | 47261970 | T | C | rs9526201 | 0.289 | -0.29[/]-0.127 | -0.199[/]-0.213 | -0.117[/]0.166 | -0.872[/]-1.008 | -0.613[/]-0.902 |
| 13 | rs1570621 | 47170118 | T | C | rs9526201 | 0.286 | -0.581[/]-0.295 | -0.3[/]-0.229 | -0.449[/]-0.26 | -0.477[/]-0.345 | -0.591[/]-0.451 |
| 13 | rs7330387 | 47097802 | T | C | rs9526201 | 0.286 | -1.479[/]-1.225 | -0.959[/]-0.674 | -1.121[/]-0.875 | -1.382[/]-1.131 | -1.23[/]-0.972 |
| 13 | rs7993865 | 47242202 | T | A | rs9526201 | 0.286 | -0.619[/]-0.885 | -1.252[/]-1.267 | -0.43[/]-0.52 | -1.376[/]-1.347 | -1.042[/]-1.329 |
| 13 | rs9534449 | 47218059 | C | T | rs9526201 | 0.285 | -1.656[/]-1.679 | -1.825[/]-1.54 | -1.55[/]-1.66 | -1.177[/]-1.228 | -1.938[/]-2.074 |
| 13 | rs7984083 | 47245745 | G | A | rs9526201 | 0.284 | -0.113[/]-0.396 | -0.462[/]-0.718 | -0.383[/]-0.354 | -0.586[/]-0.505 | -0.874[/]-0.619 |
| 13 | rs7996004 | 47093502 | G | T | rs9526201 | 0.284 | 0.37[/]0.41 | 0.274[/]0.181 | 0.115[/]-0.169 | -0.358[/]-0.557 | -0.524[/]-0.551 |
| 13 | rs842406 | 47230194 | C | T | rs9526201 | 0.284 | 0.761[/]0.476 | 1.06[/]0.972 | 0.56[/]0.418 | 0.706[/]0.443 | 0.908[/]0.654 |
| 13 | rs9534448 | 47212961 | G | A | rs9526201 | 0.281 | -1.017[/]-0.949 | -1.192[/]-0.911 | -1.359[/]-1.313 | -0.851[/]-0.827 | -0.935[/]-0.957 |
| 13 | rs842368 | 47251712 | G | A | rs9526201 | 0.279 | -2.256[/]-2.179 | -1.593[/]-1.585 | -1.572[/]-1.696 | -0.776[/]-1.055 | -1.854[/]-1.79 |
| 13 | rs4245339 | 47244223 | T | G | rs9526201 | 0.278 | -0.653[/]-0.375 | -0.805[/]-0.861 | -0.833[/]-0.822 | -0.251[/]-0.101 | -0.328[/]-0.105 |
| 13 | rs842372 | 47267979 | G | A | rs9526201 | 0.278 | -0.178[/]0.1 | -0.362[/]-0.293 | -0.358[/]-0.199 | -0.104[/]-0.036 | -0.397[/]-0.279 |
| 13 | rs11619504 | 47071148 | A | G | rs9526201 | 0.275 | -0.792[/]-0.837 | -1.573[/]-1.589 | -0.934[/]-0.925 | -0.501[/]-0.226 | -0.426[/]-0.307 |
| 13 | rs842400 | 47271875 | G | A | rs9526201 | 0.275 | -1.007[/]-0.889 | -1.662[/]-1.655 | -1.645[/]-1.542 | -0.606[/]-0.598 | -0.802[/]-0.527 |
| 13 | rs11313499 | 47220090 | AC | A | rs9526201 | 0.274 | -1.954[/]-1.806 | -2.161[/]-2.182 | -2.433[/]-2.429 | -0.472[/]-0.285 | -1.247[/]-0.973 |
| 13 | rs1467605 | 47211861 | A | C | rs9526201 | 0.271 | -0.449[/]-0.206 | -0.463[/]-0.258 | -0.961[/]-0.724 | -0.43[/]-0.291 | -0.84[/]-0.569 |
| 13 | rs912428 | 47167903 | A | G | rs9526201 | 0.271 | -0.523[/]-0.266 | -1.078[/]-1.054 | -0.962[/]-0.692 | -1.421[/]-1.522 | -0.611[/]-0.413 |
| 13 | rs2005053 | 47152942 | T | C | rs9526201 | 0.266 | -0.035[/]-0.3 | 0.074[/]-0.058 | -0.951[/]-0.863 | -1.817[/]-1.788 | -1.004[/]-1.076 |
| 13 | rs7999058 | 47216812 | C | G | rs9526201 | 0.265 | -1.276[/]-1.149 | -0.993[/]-1.064 | -0.772[/]-0.541 | -0.63[/]-0.644 | -1.075[/]-0.811 |
| 13 | rs842365 | 47252379 | G | A | rs9526201 | 0.263 | -2.293[/]-2.249 | -1.18[/]-1.283 | -1.801[/]-1.929 | -1.693[/]-1.609 | -1.614[/]-1.35 |
| 13 | rs12427491 | 47205810 | C | T | rs9526201 | 0.259 | -1.874[/]-2.01 | -0.668[/]-0.927 | -1.342[/]-1.353 | -0.936[/]-0.935 | -1.396[/]-1.604 |
| 13 | rs2794661 | 47323718 | T | C | rs9526201 | 0.259 | -1.178[/]-1.16 | -0.573[/]-0.413 | -1.175[/]-0.996 | -2.077[/]-1.817 | -1.069[/]-1.007 |
| 13 | rs9534442 | 47197754 | C | T | rs9526201 | 0.259 | 0.003[/]-0.256 | 0.803[/]0.603 | 0.539[/]0.458 | 0.466[/]0.26 | -0.268[/]-0.319 |
| 13 | rs4942550 | 47104680 | A | G | rs9526201 | 0.258 | -0.623[/]-0.508 | -0.43[/]-0.261 | -0.716[/]-0.61 | -0.499[/]-0.241 | -0.25[/]-0.041 |
| 13 | rs1773126 | 47225326 | G | A | rs9526201 | 0.257 | 0.69[/]0.509 | 0.261[/]0.005 | -0.118[/]-0.166 | 0.376[/]0.21 | 0.137[/]0.386 |
| 13 | rs9316216 | 47193442 | C | T | rs9526201 | 0.256 | -2.217[/]-2.198 | -2.212[/]-2.355 | -2.249[/]-2.345 | -2.108[/]-2.158 | -2.169[/]-2.425 |
| 13 | rs1747228 | 47221012 | T | C | rs9526201 | 0.255 | -3.424[/]-3.522 | -3.158[/]-3.066 | -2.948[/]-2.693 | -2.642[/]-2.483 | -2.592[/]-2.501 |
| 13 | rs9567717 | 47241801 | C | T | rs9526201 | 0.255 | -2.393[/]-2.648 | -1.425[/]-1.568 | -1.851[/]-1.986 | -1.709[/]-1.772 | -2.192[/]-2.228 |
| 13 | rs842367 | 47251737 | A | T | rs9526201 | 0.254 | -2.441[/]-2.582 | -1.831[/]-1.827 | -1.66[/]-1.641 | -1.015[/]-0.866 | -2.119[/]-1.864 |
| 13 | rs4942555 | 47174469 | A | G | rs9526201 | 0.253 | -1.08[/]-0.829 | -0.031[/]0.17 | -0.801[/]-0.679 | -1.56[/]-1.813 | -1.249[/]-1.213 |
| 13 | rs61948157 | 47095213 | T | C | rs9526201 | 0.253 | -1.744[/]-1.53 | -2.017[/]-1.997 | -2.213[/]-2.035 | -1.663[/]-1.41 | -2.526[/]-2.454 |
| 13 | rs842404 | 47270748 | C | G | rs9526201 | 0.252 | -0.454[/]-0.476 | -0.927[/]-0.79 | -0.873[/]-0.77 | -0.516[/]-0.768 | -0.173[/]-0.267 |
| 13 | rs11619988 | 47289240 | C | T | rs9526201 | 0.251 | -1.551[/]-1.678 | -1.395[/]-1.557 | -1.264[/]-1.515 | -0.849[/]-0.879 | -1.161[/]-1.234 |
| 13 | rs9526210 | 47223777 | C | T | rs9526201 | 0.251 | -1.16[/]-1.411 | -0.792[/]-0.618 | -0.845[/]-0.862 | -1.832[/]-1.796 | -1.296[/]-1.222 |
| 13 | rs4942561 | 47209347 | G | T | rs9526201 | 0.249 | -0.178[/]0.071 | -0.353[/]-0.226 | -0.151[/]0.008 | 0.12[/]0.329 | -0.174[/]0.008 |
| 13 | rs842408 | 47228097 | T | A | rs9526201 | 0.249 | -1.311[/]-1.115 | -1.568[/]-1.411 | -1.233[/]-0.984 | -0.571[/]-0.442 | -1.358[/]-1.235 |
| 13 | rs12429799 | 47294903 | T | C | rs9526201 | 0.248 | -1.474[/]-1.357 | -0.456[/]-0.209 | -0.71[/]-0.557 | -1.387[/]-1.147 | -1.242[/]-1.087 |
| 13 | rs493582 | 47075711 | C | T | rs9526201 | 0.248 | -1.785[/]-1.803 | -1.479[/]-1.539 | -1.372[/]-1.516 | -1.406[/]-1.446 | -1.415[/]-1.663 |
| 13 | rs7319949 | 47148843 | T | G | rs9526201 | 0.243 | -0.595[/]-0.706 | 0.164[/]0.315 | -0.567[/]-0.496 | -0.593[/]-0.836 | -0.905[/]-0.802 |
| 13 | rs9534434 | 47172726 | C | A | rs9526201 | 0.243 | -0.804[/]-0.849 | -0.833[/]-0.75 | -1.394[/]-1.259 | -0.511[/]-0.753 | -0.802[/]-0.783 |
| 13 | rs1467606 | 47211758 | T | C | rs9526201 | 0.241 | 0.152[/]0.301 | -0.509[/]-0.305 | -1.149[/]-0.909 | -0.733[/]-0.518 | -1.009[/]-0.898 |
| 13 | rs4942563 | 47210802 | C | T | rs9526201 | 0.24 | -0.017[/]-0.013 | -1.034[/]-1.274 | -1.026[/]-1.146 | -1.581[/]-1.604 | -1.38[/]-1.412 |
| 13 | rs7333028 | 47238684 | T | C | rs9526201 | 0.24 | -0.52[/]-0.421 | -0.106[/]0.134 | -0.789[/]-0.989 | -0.574[/]-0.505 | -0.765[/]-0.815 |
| 13 | rs4942557 | 47183068 | G | A | rs9526201 | 0.239 | -1.363[/]-1.247 | -0.972[/]-0.941 | -1.392[/]-1.394 | -0.763[/]-0.592 | -1.477[/]-1.238 |
| 13 | rs9534416 | 47096271 | C | T | rs9526201 | 0.239 | -0.567[/]-0.525 | -0.157[/]-0.207 | -0.299[/]-0.37 | 0.1[/]0.048 | 0.3[/]0.061 |
| 13 | rs9567710 | 47200731 | C | A | rs9526201 | 0.239 | -0.864[/]-1.026 | -1.445[/]-1.684 | -1.368[/]-1.561 | -1.389[/]-1.607 | -1.663[/]-1.788 |
| 13 | rs1570620 | 47201874 | C | T | rs9526201 | 0.238 | -0.525[/]-0.762 | -0.644[/]-0.709 | -0.762[/]-0.636 | -0.329[/]-0.266 | -0.395[/]-0.416 |
| 13 | rs67760743 | 47083879 | T | TC | rs9526201 | 0.237 | -0.921[/]-0.891 | -1.039[/]-1.156 | -1.251[/]-1.152 | -1.339[/]-1.207 | -1.881[/]-1.645 |
| 13 | rs842412 | 47226506 | T | C | rs9526201 | 0.237 | -1.767[/]-1.563 | -1.615[/]-1.852 | -1.631[/]-1.525 | -1.13[/]-1.123 | -1.937[/]-1.896 |
| 13 | rs1570974 | 47108253 | G | A | rs9526201 | 0.236 | 0.23[/]-0.005 | -0.705[/]-0.691 | -0.47[/]-0.47 | -0.639[/]-0.775 | 0.016[/]-0.108 |
| 13 | rs7337999 | 47143284 | C | T | rs9526201 | 0.236 | -0.666[/]-0.783 | -0.396[/]-0.453 | -0.369[/]-0.546 | -0.364[/]-0.539 | -0.758[/]-0.993 |
| 13 | rs6561323 | 47185601 | A | G | rs9526201 | 0.233 | -1.984[/]-1.829 | -1.63[/]-1.397 | -1.443[/]-1.365 | -1.786[/]-1.7 | -1.472[/]-1.399 |
| 13 | rs1570622 | 47169092 | G | A | rs9526201 | 0.232 | -1.182[/]-1.413 | 0.535[/]0.595 | -0.012[/]-0.097 | -1.06[/]-1.11 | -0.801[/]-0.824 |
| 13 | rs7994173 | 47107188 | A | T | rs9526201 | 0.23 | -1.34[/]-1.378 | -0.836[/]-0.89 | -1.483[/]-1.455 | -1.462[/]-1.233 | -1.229[/]-1.201 |
| 13 | rs7333406 | 47299045 | G | T | rs9526201 | 0.228 | -1.357[/]-1.336 | -1.216[/]-1.444 | -1.095[/]-1.24 | -1.582[/]-1.49 | -1.173[/]-1.192 |
| 13 | rs767851 | 47233744 | C | T | rs9526201 | 0.226 | -0.767[/]-0.603 | -1.088[/]-1.014 | -0.74[/]-0.764 | -0.87[/]-1.095 | -1.582[/]-1.67 |
| 13 | rs4942549 | 47099664 | C | T | rs9526201 | 0.225 | -1.295[/]-1.52 | -1.842[/]-1.909 | -1.963[/]-2.006 | -1.893[/]-1.985 | -2.197[/]-2.325 |
| 13 | rs4942567 | 47218834 | A | G | rs9526201 | 0.224 | -1.457[/]-1.365 | -1.542[/]-1.676 | -1.81[/]-1.817 | -1.486[/]-1.377 | -2.321[/]-2.097 |
| 13 | rs9595526 | 47314115 | T | A | rs9526201 | 0.222 | -0.682[/]-0.801 | -1.554[/]-1.776 | -0.945[/]-1.157 | -0.762[/]-0.619 | -1.024[/]-1.229 |
| 13 | rs7987387 | 47241022 | T | A | rs9526201 | 0.22 | -0.269[/]-0.05 | -0.28[/]-0.145 | -0.379[/]-0.307 | -0.535[/]-0.58 | -1.039[/]-0.838 |
| 13 | rs79884924 | 47201607 | AT | A | rs9526201 | 0.22 | -1.416[/]-1.362 | -0.855[/]-0.635 | -1.244[/]-1.105 | -0.971[/]-0.958 | -1.39[/]-1.472 |
| 13 | rs7995870 | 47172275 | T | G | rs9526201 | 0.219 | -1.948[/]-1.729 | -1.282[/]-1.223 | -1.744[/]-1.665 | -1.293[/]-1.28 | -2.039[/]-1.966 |
| 13 | rs9526214 | 47237213 | T | C | rs9526201 | 0.219 | -0.757[/]-0.78 | -0.885[/]-0.666 | -0.531[/]-0.448 | -0.455[/]-0.401 | -0.772[/]-0.837 |
| 13 | rs7986508 | 47241289 | C | T | rs9526201 | 0.217 | -1.31[/]-1.399 | -1.399[/]-1.386 | -1.136[/]-1.128 | -0.625[/]-0.584 | -1.196[/]-0.979 |
| 13 | rs9316217 | 47209806 | G | C | rs9526201 | 0.217 | -2.916[/]-3.099 | -2.824[/]-2.766 | -2.791[/]-2.972 | -2.632[/]-2.799 | -2.751[/]-2.968 |
| 13 | rs34704437 | 47226159 | AC | A | rs9526201 | 0.215 | -2.059[/]-2.101 | -1.931[/]-2.145 | -1.81[/]-1.817 | -0.864[/]-0.953 | -1.777[/]-1.776 |
| 13 | rs9316206 | 47086579 | A | C | rs9526201 | 0.212 | -1.031[/]-1.243 | -0.703[/]-0.744 | -1.062[/]-1.088 | -1.265[/]-1.349 | -1.106[/]-1.092 |
| 13 | rs842363 | 47253270 | C | A | rs9526201 | 0.211 | -0.255[/]-0.421 | -0.897[/]-1.001 | -1.202[/]-1.295 | -0.26[/]-0.471 | -0.484[/]-0.615 |
| 13 | rs6561325 | 47265887 | C | T | rs9526201 | 0.21 | -1.745[/]-1.829 | -1.821[/]-1.922 | -1.636[/]-1.846 | -1.776[/]-1.74 | -2.445[/]-2.437 |
| 13 | rs837 | 47224772 | G | A | rs9526201 | 0.21 | -0.929[/]-0.958 | 0.192[/]0.381 | -0.49[/]-0.486 | -1.06[/]-1.252 | -0.453[/]-0.664 |
| 13 | rs9534450 | 47218559 | A | T | rs9526201 | 0.21 | -0.285[/]-0.495 | -0.804[/]-0.779 | -1.085[/]-1.006 | -0.268[/]-0.388 | -1.008[/]-0.906 |
| 13 | rs842384 | 47257864 | C | T | rs9526201 | 0.208 | -1.459[/]-1.346 | -1.536[/]-1.665 | -1.203[/]-1.169 | -0.892[/]-0.749 | -1.237[/]-1.029 |
| 13 | rs11273761 | 47095694 | G | GCATCTCAGTTTTCTCATAA | rs9526201 | 0.207 | -1.654[/]-1.777 | -1.825[/]-2.033 | -1.6[/]-1.715 | -1.891[/]-1.888 | -1.79[/]-1.961 |
| 13 | rs145089234 | 47224877 | AG | A | rs9526201 | 0.206 | -1.061[/]-0.972 | 0.197[/]0.403 | -0.131[/]0.002 | -1.036[/]-0.948 | -0.548[/]-0.406 |
| 13 | rs2405413 | 47113763 | T | C | rs9526201 | 0.206 | -0.938[/]-0.732 | -0.374[/]-0.315 | -0.467[/]-0.388 | -0.92[/]-0.774 | -0.817[/]-0.698 |
| 13 | rs8000003 | 47202798 | G | A | rs9526201 | 0.206 | -1.072[/]-1.048 | -0.942[/]-0.946 | -1.146[/]-1.065 | -1.209[/]-1.021 | -1.198[/]-0.993 |
| 13 | rs9526207 | 47201226 | C | T | rs9526201 | 0.204 | -1.248[/]-1.23 | -1.163[/]-1.356 | -1.451[/]-1.417 | -0.356[/]-0.56 | -1.17[/]-1.228 |
| 13 | rs9526189 | 47097111 | C | T | rs9526201 | 0.203 | -1.393[/]-1.527 | -0.968[/]-0.912 | -0.858[/]-1.001 | -1.535[/]-1.633 | -1.224[/]-1.426 |
| 13 | rs41284185 | 47243196 | G | C | rs9526201 | 0.202 | -0.018[/]0.06 | -0.546[/]-0.674 | -0.58[/]-0.522 | -0.753[/]-0.886 | 0.264[/]0.062 |
| 13 | rs4941565 | 47242912 | T | G | rs9526201 | 0.202 | -1.557[/]-1.758 | -1.256[/]-1.348 | -1.259[/]-1.236 | -1.278[/]-1.249 | -1.117[/]-1.257 |
| 13 | rs4942565 | 47214373 | C | A | rs9526201 | 0.202 | -1.125[/]-1.188 | -1.382[/]-1.584 | -1.021[/]-1.14 | -0.927[/]-0.996 | -1.115[/]-1.218 |
| 13 | rs12429724 | 47294088 | A | G | rs9526201 | 0.201 | -2.926[/]-2.814 | -2.818[/]-2.795 | -3.044[/]-2.856 | -2.14[/]-2.341 | -2.822[/]-2.853 |
| 13 | rs1773132 | 47221109 | G | A | rs9526201 | 0.201 | -3.53[/]-3.731 | -3.308[/]-3.281 | -2.88[/]-3.033 | -2.819[/]-2.814 | -2.214[/]-2.406 |
| 13 | rs970404 | 47219427 | A | G | rs9526201 | 0.201 | -2.4[/]-2.601 | -1.89[/]-1.831 | -1.813[/]-1.613 | -1.391[/]-1.37 | -1.237[/]-1.33 |
| 13 | rs1886967 | 47098648 | A | T | rs9526201 | 0.199 | -0.76[/]-0.909 | -0.308[/]-0.216 | -0.414[/]-0.215 | 0.426[/]0.435 | 0.298[/]0.321 |
| 13 | rs1008849 | 47184932 | C | T | rs9526201 | 0.198 | -0.655[/]-0.729 | -0.327[/]-0.236 | -0.699[/]-0.501 | -1.201[/]-1.096 | -0.803[/]-0.713 |
| 13 | rs1326113 | 47100585 | T | C | rs9526201 | 0.196 | 0.144[/]0.25 | -0.606[/]-0.681 | -0.254[/]-0.078 | -0.557[/]-0.753 | -0.717[/]-0.677 |
| 13 | rs6561322 | 47163185 | T | A | rs9526201 | 0.192 | -2.102[/]-1.91 | -1.844[/]-1.822 | -2.505[/]-2.598 | -1.691[/]-1.814 | -2.32[/]-2.393 |
| 13 | rs7331562 | 47298850 | T | C | rs9526201 | 0.192 | -1.361[/]-1.33 | -0.889[/]-0.819 | -1.098[/]-1.29 | -0.638[/]-0.699 | -0.612[/]-0.665 |
| 13 | rs545742 | 47083369 | C | T | rs9526201 | 0.191 | -1.037[/]-1.229 | -0.166[/]-0.273 | -0.342[/]-0.509 | -1.274[/]-1.285 | -0.859[/]-0.737 |
| 13 | rs6561319 | 47112120 | C | A | rs9526201 | 0.19 | -2.142[/]-2.006 | -1.789[/]-1.89 | -1.769[/]-1.655 | -1.673[/]-1.65 | -1.952[/]-1.762 |
| 13 | rs842390 | 47277228 | A | G | rs9526201 | 0.19 | 0.026[/]0.216 | -0.306[/]-0.153 | 0.128[/]0.285 | -0.544[/]-0.556 | 0.164[/]0.318 |
| 13 | rs754106 | 47152655 | C | T | rs9526201 | 0.189 | -0.601[/]-0.574 | -1.461[/]-1.381 | -1.341[/]-1.373 | -0.803[/]-0.992 | -0.862[/]-0.777 |
| 13 | rs1535791 | 47165458 | T | C | rs9526201 | 0.188 | -1.379[/]-1.283 | -1.478[/]-1.29 | -1.009[/]-0.906 | -0.774[/]-0.704 | -1.631[/]-1.472 |
| 13 | rs2147162 | 47270023 | C | G | rs9526201 | 0.188 | -1.207[/]-1.395 | -0.147[/]-0.055 | -1.217[/]-1.208 | -1.674[/]-1.548 | -2.081[/]-2.129 |
| 13 | rs9567720 | 47268157 | C | A | rs9526201 | 0.187 | -1.038[/]-0.851 | -0.703[/]-0.804 | -0.978[/]-1.044 | -1.126[/]-1.206 | -0.814[/]-0.855 |
| 13 | rs842409 | 47227961 | G | T | rs9526201 | 0.186 | -1.756[/]-1.82 | -2.027[/]-2.083 | -1.766[/]-1.952 | -0.855[/]-0.932 | -1.575[/]-1.755 |
| 13 | rs55705503 | 47276339 | G | GTTAT | rs9526201 | 0.183 | -1.954[/]-2.081 | -1.219[/]-1.402 | -1.898[/]-1.797 | -1.821[/]-1.677 | -2.853[/]-2.842 |
| 13 | rs1886964 | 47175084 | C | T | rs9526201 | 0.181 | 0.656[/]0.496 | 0.633[/]0.732 | 0.3[/]0.118 | 0.096[/]-0.071 | -0.492[/]-0.493 |
| 13 | rs9526218 | 47267492 | C | G | rs9526201 | 0.181 | -0.466[/]-0.613 | -0.714[/]-0.895 | -1.334[/]-1.42 | -1.526[/]-1.493 | -0.954[/]-0.871 |
| 13 | rs35283972 | 47288513 | G | A | rs9526201 | 0.178 | -1.87[/]-1.938 | -1.283[/]-1.461 | -1.812[/]-1.647 | -1.377[/]-1.427 | -1.597[/]-1.497 |
| 13 | rs4941563 | 47121510 | C | A | rs9526201 | 0.178 | -0.802[/]-0.922 | -0.518[/]-0.362 | -0.691[/]-0.668 | -0.558[/]-0.707 | -0.448[/]-0.626 |
| 13 | rs7330453 | 47171755 | G | A | rs9526201 | 0.178 | -2.383[/]-2.44 | -1.844[/]-1.762 | -2.085[/]-2.263 | -1.214[/]-1.254 | -1.943[/]-1.848 |
| 13 | rs6561312 | 47098806 | G | A | rs9526201 | 0.176 | -0.202[/]-0.209 | -0.561[/]-0.737 | -0.522[/]-0.585 | -0.321[/]-0.308 | 0.026[/]0.148 |
| 13 | rs12584117 | 47090614 | C | G | rs9526201 | 0.174 | -1.163[/]-1.292 | -1.337[/]-1.231 | -1.065[/]-1.179 | -1.364[/]-1.538 | -1.167[/]-1.075 |
| 13 | rs842411 | 47227238 | C | T | rs9526201 | 0.174 | -1.344[/]-1.366 | -0.874[/]-0.929 | -2[/]-1.928 | -1.201[/]-1.141 | -0.56[/]-0.386 |
| 13 | rs1773118 | 47221640 | T | G | rs9526201 | 0.173 | -0.829[/]-0.955 | -1.183[/]-1.02 | -1.076[/]-1.215 | -1.703[/]-1.572 | -0.697[/]-0.87 |
| 13 | rs5803362 | 47203441 | GT | G | rs9526201 | 0.173 | -1.862[/]-2.001 | -1.074[/]-1.18 | -1.311[/]-1.422 | -1.261[/]-1.303 | -1.382[/]-1.555 |
| 13 | rs8001837 | 47212204 | G | A | rs9526201 | 0.171 | -1.6[/]-1.708 | -1.438[/]-1.525 | -1.362[/]-1.325 | -0.618[/]-0.592 | -1.058[/]-1.23 |
| 13 | rs842410 | 47227450 | G | A | rs9526201 | 0.171 | -0.48[/]-0.54 | -0.946[/]-0.804 | -1.313[/]-1.142 | -0.636[/]-0.683 | -0.62[/]-0.575 |
| 13 | rs9567707 | 47175435 | T | C | rs9526201 | 0.171 | -0.245[/]-0.132 | -1.36[/]-1.531 | -2.076[/]-2.044 | -1.278[/]-1.244 | -1.361[/]-1.311 |
| 13 | rs4942547 | 47097227 | C | T | rs9526201 | 0.17 | -1.136[/]-1.08 | -1.072[/]-0.907 | -1.454[/]-1.285 | -1.544[/]-1.528 | -1.559[/]-1.397 |
| 13 | rs639360 | 47085132 | C | T | rs9526201 | 0.169 | -1.234[/]-1.144 | -1.559[/]-1.391 | -1.427[/]-1.4 | -1.846[/]-1.936 | -1.783[/]-1.748 |
| 13 | rs7331563 | 47298857 | T | C | rs9526201 | 0.169 | -1.233[/]-1.329 | -0.983[/]-0.814 | -1.173[/]-1.237 | -0.676[/]-0.667 | -0.616[/]-0.655 |
| 13 | rs61949287 | 47177105 | A | G | rs9526201 | 0.166 | -2.249[/]-2.213 | -1.653[/]-1.656 | -2.062[/]-2.228 | -1.786[/]-1.817 | -1.459[/]-1.411 |
| 13 | rs6561321 | 47161560 | C | A | rs9526201 | 0.166 | -0.133[/]-0.088 | -0.699[/]-0.533 | -1.04[/]-0.98 | -0.362[/]-0.396 | -0.842[/]-0.824 |
| 13 | rs706620 | 47278093 | C | T | rs9526201 | 0.166 | -2.229[/]-2.175 | -1.173[/]-1.167 | -1.195[/]-1.162 | -1.109[/]-1.275 | -1.913[/]-2.075 |
| 13 | rs2761950 | 47085889 | A | G | rs9526201 | 0.164 | -1.597[/]-1.504 | -2.422[/]-2.423 | -2.476[/]-2.312 | -1.688[/]-1.622 | -1.336[/]-1.187 |
| 13 | rs1747223 | 47224681 | A | G | rs9526201 | 0.163 | -1.211[/]-1.206 | -0.882[/]-0.719 | -1.214[/]-1.058 | -1.393[/]-1.377 | -0.173[/]-0.143 |
| 13 | rs112416609 | 47260706 | A | AAGAG | rs9526201 | 0.162 | -1.596[/]-1.526 | -1.514[/]-1.403 | -1.298[/]-1.343 | -1.422[/]-1.585 | -1.418[/]-1.375 |
| 13 | rs9567690 | 47087936 | A | T | rs9526201 | 0.162 | -1.126[/]-1.226 | -0.917[/]-0.942 | -0.934[/]-0.771 | -0.956[/]-0.929 | -1.564[/]-1.487 |
| 13 | rs4942570 | 47250904 | A | G | rs9526201 | 0.161 | -1.554[/]-1.505 | -1.082[/]-1.244 | -1.694[/]-1.673 | -0.441[/]-0.314 | -1.077[/]-0.933 |
| 13 | rs842407 | 47228730 | C | G | rs9526201 | 0.159 | -2.079[/]-2.191 | -2.335[/]-2.351 | -1.7[/]-1.708 | -1.903[/]-1.766 | -2.224[/]-2.383 |
| 13 | rs9595479 | 47083878 | T | G | rs9526201 | 0.159 | -0.911[/]-0.861 | -1.029[/]-0.948 | -1.247[/]-1.245 | -1.338[/]-1.178 | -1.876[/]-1.735 |
| 13 | rs11618695 | 47290530 | C | T | rs9526201 | 0.155 | -1.863[/]-1.917 | -0.856[/]-0.956 | -1.569[/]-1.653 | -2.256[/]-2.101 | -1.941[/]-1.954 |
| 13 | rs842398 | 47274639 | G | T | rs9526201 | 0.155 | -0.317[/]-0.429 | -0.787[/]-0.711 | -0.752[/]-0.907 | -0.734[/]-0.684 | -1.141[/]-1.178 |
| 13 | rs9526188 | 47096658 | C | T | rs9526201 | 0.155 | -0.818[/]-0.973 | -0.125[/]-0.218 | -0.228[/]-0.211 | -0.598[/]-0.565 | -0.585[/]-0.732 |
| 13 | rs564275357 | 47102750 | GAAAAA | G | rs9526201 | 0.153 | -1.496[/]-1.566 | -1.248[/]-1.334 | -1.182[/]-1.276 | -1.405[/]-1.537 | -1.622[/]-1.775 |
| 13 | rs4942553 | 47155975 | A | G | rs9526201 | 0.151 | -1.726[/]-1.576 | -1.628[/]-1.604 | -0.927[/]-0.944 | -0.838[/]-0.785 | -0.519[/]-0.657 |
| 13 | rs568025 | 47074083 | A | C | rs9526201 | 0.151 | -0.947[/]-0.839 | -0.993[/]-0.842 | -0.951[/]-0.882 | -0.906[/]-0.958 | -1.41[/]-1.406 |
| 13 | rs34944979 | 47081841 | AG | A | rs9526201 | 0.15 | -1.174[/]-1.196 | -1.771[/]-1.699 | -1.502[/]-1.353 | -1.381[/]-1.382 | -1.475[/]-1.359 |
| 13 | rs4942545 | 47095594 | G | A | rs9526201 | 0.15 | -0.211[/]-0.243 | -1.425[/]-1.571 | -0.708[/]-0.741 | -1.025[/]-1.164 | -0.962[/]-1.112 |
| 13 | rs7986104 | 47206196 | C | G | rs9526201 | 0.149 | -2.338[/]-2.465 | -2.579[/]-2.71 | -2.597[/]-2.741 | -2.304[/]-2.452 | -2.434[/]-2.536 |
| 13 | rs10609400 | 47159105 | CAT | C | rs9526201 | 0.148 | -1.157[/]-1.099 | -0.344[/]-0.197 | -0.525[/]-0.527 | -1.184[/]-1.082 | -0.794[/]-0.762 |
| 13 | rs61429104 | 47264206 | G | GA | rs9526201 | 0.147 | -0.949[/]-0.896 | -1.375[/]-1.229 | -0.45[/]-0.357 | -0.991[/]-0.907 | -0.544[/]-0.443 |
| 13 | rs7989491 | 47219006 | A | G | rs9526201 | 0.146 | -1.222[/]-1.368 | -1.156[/]-1.224 | -2.046[/]-1.948 | -1.15[/]-1.079 | -1.895[/]-1.844 |
| 13 | rs6561314 | 47099042 | C | T | rs9526201 | 0.145 | -0.608[/]-0.753 | -0.852[/]-0.982 | -0.864[/]-0.82 | -0.402[/]-0.433 | -0.541[/]-0.511 |
| 13 | rs9526219 | 47267499 | A | G | rs9526201 | 0.145 | -0.48[/]-0.335 | -0.773[/]-0.75 | -1.393[/]-1.263 | -1.567[/]-1.515 | -0.936[/]-0.951 |
| 13 | rs842402 | 47270850 | A | C | rs9526201 | 0.143 | -1.685[/]-1.699 | -2.093[/]-2.143 | -1.979[/]-2.052 | -1.659[/]-1.677 | -1.704[/]-1.561 |
| 13 | rs7993628 | 47242065 | T | C | rs9526201 | 0.142 | -1.561[/]-1.603 | -1.881[/]-1.962 | -1.873[/]-1.732 | -2.204[/]-2.226 | -0.851[/]-0.717 |
| 13 | rs7336742 | 47069292 | A | G | rs9526201 | 0.139 | -0.529[/]-0.662 | -0.841[/]-0.857 | -0.898[/]-0.9 | -0.684[/]-0.545 | -0.901[/]-0.896 |
| 13 | rs1747226 | 47221847 | G | T | rs9526201 | 0.137 | 0.179[/]0.282 | 0.776[/]0.639 | 0.777[/]0.803 | 0.001[/]-0.136 | 0.474[/]0.343 |
| 13 | rs35433062 | 47188247 | CT | C | rs9526201 | 0.137 | -1.073[/]-1.018 | -0.618[/]-0.559 | -1.076[/]-0.939 | -1.208[/]-1.202 | -0.615[/]-0.538 |
| 13 | rs9316219 | 47212520 | C | T | rs9526201 | 0.137 | -0.591[/]-0.665 | -0.099[/]-0.234 | -0.492[/]-0.599 | -0.528[/]-0.542 | -0.915[/]-1.052 |
| 13 | rs6561315 | 47099426 | T | G | rs9526201 | 0.135 | -1.978[/]-1.87 | -1.462[/]-1.52 | -1.898[/]-1.891 | -1.434[/]-1.379 | -1.7[/]-1.565 |
| 13 | rs6561316 | 47099561 | A | T | rs9526201 | 0.135 | -1.127[/]-1.173 | -1.218[/]-1.204 | -1.683[/]-1.734 | -1.407[/]-1.541 | -1.709[/]-1.669 |
| 13 | rs842241 | 47067841 | T | C | rs9526201 | 0.135 | -2.514[/]-2.474 | -2.008[/]-1.953 | -2.001[/]-2.052 | -1.779[/]-1.809 | -2.625[/]-2.489 |
| 13 | rs6561313 | 47098897 | C | A | rs9526201 | 0.134 | -0.192[/]-0.243 | -1.033[/]-0.908 | -0.811[/]-0.823 | -0.646[/]-0.633 | -0.46[/]-0.325 |
| 13 | rs1007171 | 47106226 | T | G | rs9526201 | 0.133 | -0.314[/]-0.193 | -0.174[/]-0.23 | -0.396[/]-0.456 | 0.004[/]0.136 | -0.321[/]-0.427 |
| 13 | rs11148012 | 47231744 | C | T | rs9526201 | 0.132 | -1.612[/]-1.594 | -1.157[/]-1.149 | -1.769[/]-1.74 | -1.779[/]-1.647 | -1.407[/]-1.311 |
| 13 | rs3832915 | 47279680 | A | AT | rs9526201 | 0.132 | -1.148[/]-1.237 | -0.638[/]-0.585 | -0.86[/]-0.956 | -0.463[/]-0.336 | -1.038[/]-0.906 |
| 13 | rs9534445 | 47206989 | C | T | rs9526201 | 0.132 | -1.969[/]-2.011 | -1.712[/]-1.58 | -2.384[/]-2.275 | -1.549[/]-1.497 | -1.346[/]-1.419 |
| 13 | rs2405412 | 47070609 | C | T | rs9526201 | 0.131 | -2.024[/]-2.082 | -1.341[/]-1.434 | -1.729[/]-1.853 | -1.569[/]-1.473 | -1.604[/]-1.735 |
| 13 | rs8001925 | 47239853 | G | A | rs9526201 | 0.131 | 0.033[/]-0.049 | -0.26[/]-0.31 | -0.213[/]-0.154 | -0.994[/]-1.057 | -1.015[/]-1.146 |
| 13 | rs842373 | 47263676 | C | A | rs9526201 | 0.13 | -1.83[/]-1.791 | -1.057[/]-1.032 | -1.405[/]-1.275 | -1.001[/]-1.034 | -1.201[/]-1.137 |
| 13 | rs7992307 | 47242104 | C | G | rs9526201 | 0.129 | -1.147[/]-1.204 | -1.433[/]-1.316 | -1.205[/]-1.133 | -2.046[/]-2.025 | -0.747[/]-0.876 |
| 13 | rs143636430 | 47242857 | CGGTGGCGG | C | rs9526201 | 0.127 | -1.069[/]-1.014 | -0.864[/]-0.83 | -0.565[/]-0.617 | -0.74[/]-0.776 | -0.803[/]-0.675 |
| 13 | rs3742271 | 47242927 | G | C | rs9526201 | 0.127 | -1.709[/]-1.629 | -1.379[/]-1.252 | -1.508[/]-1.563 | -1.398[/]-1.328 | -1.308[/]-1.323 |
| 13 | rs1535793 | 47154966 | G | A | rs9526201 | 0.124 | -0.164[/]-0.269 | -0.483[/]-0.493 | -0.694[/]-0.818 | -0.93[/]-0.958 | -0.42[/]-0.352 |
| 13 | rs17282423 | 47290217 | G | A | rs9526201 | 0.121 | -1.503[/]-1.504 | -1.677[/]-1.679 | -1.414[/]-1.386 | -1.868[/]-1.747 | -2.449[/]-2.436 |
| 13 | rs12872545 | 47304538 | T | G | rs9526201 | 0.12 | -1.127[/]-1.014 | 0.278[/]0.366 | 0.533[/]0.504 | -0.775[/]-0.814 | -0.616[/]-0.737 |
| 13 | rs9534433 | 47158041 | G | A | rs9526201 | 0.12 | -1.252[/]-1.229 | -1.489[/]-1.453 | -1.359[/]-1.444 | -0.617[/]-0.526 | -1.28[/]-1.16 |
| 13 | rs1886966 | 47094949 | T | C | rs9526201 | 0.119 | -0.931[/]-0.96 | -0.674[/]-0.629 | -0.869[/]-0.921 | -0.55[/]-0.431 | -1.284[/]-1.179 |
| 13 | rs564512645 | 47247881 | ATTTTTTT | A | rs9526201 | 0.117 | -1.43[/]-1.509 | -1.181[/]-1.297 | -1.051[/]-1.167 | -0.987[/]-1.06 | -0.99[/]-1.04 |
| 13 | rs1570975 | 47103489 | T | C | rs9526201 | 0.113 | -0.889[/]-0.821 | -0.849[/]-0.736 | -0.846[/]-0.774 | -0.689[/]-0.629 | -0.677[/]-0.637 |
| 13 | rs9526208 | 47208767 | A | G | rs9526201 | 0.112 | 0.264[/]0.228 | -0.097[/]-0.026 | 0.356[/]0.294 | -0.078[/]0.034 | -0.167[/]-0.123 |
| 13 | rs67901139 | 47233298 | GATTTATTTATTTATTT | G | rs9526201 | 0.111 | -1.725[/]-1.781 | -1.566[/]-1.596 | -1.267[/]-1.268 | -1.425[/]-1.505 | -1.032[/]-1.143 |
| 13 | rs4942571 | 47266124 | A | G | rs9526201 | 0.11 | -2.165[/]-2.157 | -2.312[/]-2.349 | -2.244[/]-2.253 | -2.392[/]-2.455 | -2.084[/]-2.193 |
| 13 | rs66625580 | 47247637 | GA | G | rs9526201 | 0.109 | -1.31[/]-1.331 | -1.943[/]-2.052 | -1.756[/]-1.81 | -1.259[/]-1.283 | -1.109[/]-1.196 |
| 13 | rs4941557 | 47080410 | A | C | rs9526201 | 0.105 | -2.228[/]-2.333 | -1.962[/]-2.019 | -1.806[/]-1.911 | -1.638[/]-1.551 | -1.647[/]-1.691 |
| 13 | rs74430721 | 47091072 | C | A | rs9526201 | 0.105 | -1.35[/]-1.258 | -0.908[/]-0.803 | -1.032[/]-0.999 | -0.388[/]-0.345 | -0.333[/]-0.244 |
| 13 | rs842377 | 47261161 | T | G | rs9526201 | 0.105 | -2.056[/]-2.062 | -1.904[/]-1.913 | -2.182[/]-2.287 | -1.661[/]-1.689 | -2.138[/]-2.121 |
| 13 | rs66820930 | 47241673 | AG | A | rs9526201 | 0.095 | -1.296[/]-1.28 | -1.045[/]-1.14 | -1.046[/]-1.053 | -1.153[/]-1.216 | -1.247[/]-1.34 |
| 13 | rs9316218 | 47210159 | C | T | rs9526201 | 0.094 | -1.878[/]-1.918 | -1.371[/]-1.465 | -1.54[/]-1.615 | -1.329[/]-1.318 | -1.517[/]-1.464 |
| 13 | rs1773128 | 47225440 | T | A | rs9526201 | 0.091 | -0.72[/]-0.786 | -0.71[/]-0.734 | -1.041[/]-1.005 | -0.313[/]-0.222 | -1.069[/]-1.147 |
| 13 | rs642732 | 47077811 | A | G | rs9526201 | 0.091 | -1.957[/]-1.911 | -1.399[/]-1.309 | -1.981[/]-1.922 | -1.574[/]-1.548 | -2.246[/]-2.181 |
| 13 | rs9534413 | 47080021 | A | G | rs9526201 | 0.088 | -1.623[/]-1.701 | -1.753[/]-1.676 | -1.424[/]-1.336 | -2.034[/]-1.969 | -1.828[/]-1.772 |
| 13 | rs148781298 | 47091071 | ACAC | A | rs9526201 | 0.087 | -1.442[/]-1.355 | -0.827[/]-0.907 | -1.003[/]-1.034 | -0.331[/]-0.386 | -0.399[/]-0.336 |
| 13 | rs139602811 | 47162731 | CTTT | C | rs9526201 | 0.085 | -1.682[/]-1.758 | -1.02[/]-1.073 | -1.215[/]-1.3 | -0.815[/]-0.807 | -0.763[/]-0.755 |
| 13 | rs572205411 | 47156783 | C | CTT | rs9526201 | 0.085 | -1.983[/]-1.915 | -1.673[/]-1.592 | -1.681[/]-1.595 | -0.946[/]-0.926 | -1.042[/]-1.022 |
| 13 | rs4941566 | 47243739 | G | A | rs9526201 | 0.084 | -1.759[/]-1.843 | -1.06[/]-0.983 | -1.371[/]-1.408 | -1.313[/]-1.317 | -1.384[/]-1.398 |
| 13 | rs7337752 | 47156855 | T | C | rs9526201 | 0.084 | -2.118[/]-2.103 | -1.91[/]-1.949 | -1.895[/]-1.967 | -1.06[/]-1.144 | -1.339[/]-1.411 |
| 13 | rs9526212 | 47225745 | A | G | rs9526201 | 0.084 | -1.345[/]-1.261 | -1.03[/]-1.106 | -1.624[/]-1.574 | -0.787[/]-0.795 | -1.296[/]-1.234 |
| 13 | rs3068686 | 47100807 | AGGTGTTT | A | rs9526201 | 0.083 | -0.069[/]0.013 | -0.319[/]-0.239 | -0.475[/]-0.411 | -0.608[/]-0.581 | -0.12[/]-0.192 |
| 13 | rs9526216 | 47243910 | A | G | rs9526201 | 0.08 | -0.653[/]-0.662 | -1.334[/]-1.362 | -1.191[/]-1.111 | -1.216[/]-1.253 | -0.756[/]-0.799 |
| 13 | rs9567686 | 47081599 | T | G | rs9526201 | 0.075 | -1.711[/]-1.641 | -1.71[/]-1.681 | -1.906[/]-1.875 | -1.776[/]-1.851 | -1.578[/]-1.635 |
| 13 | rs1012628 | 47119642 | C | T | rs9526201 | 0.073 | -1.327[/]-1.315 | -2.068[/]-2.104 | -1.462[/]-1.407 | -1.524[/]-1.58 | -2.043[/]-2.116 |
| 13 | rs113204301 | 47171940 | A | AT | rs9526201 | 0.073 | -0.895[/]-0.88 | -0.677[/]-0.605 | -1.073[/]-1 | -0.866[/]-0.83 | -0.808[/]-0.754 |
| 13 | rs1326112 | 47100498 | C | T | rs9526201 | 0.073 | -0.166[/]-0.119 | -0.294[/]-0.228 | -0.011[/]-0.033 | -0.654[/]-0.726 | -0.721[/]-0.728 |
| 13 | rs5803364 | 47231298 | AT | A | rs9526201 | 0.07 | -0.818[/]-0.829 | -0.686[/]-0.738 | -1.321[/]-1.391 | -1.516[/]-1.557 | -0.787[/]-0.823 |
| 13 | rs57803401 | 47226530 | G | GT | rs9526201 | 0.068 | -1.666[/]-1.634 | -1.43[/]-1.375 | -1.542[/]-1.48 | -0.938[/]-0.923 | -1.854[/]-1.785 |
| 13 | rs5803363 | 47211847 | G | GT | rs9526201 | 0.068 | -0.353[/]-0.351 | -0.461[/]-0.405 | -0.906[/]-0.838 | -0.413[/]-0.392 | -0.748[/]-0.701 |
| 13 | rs11483731 | 47279375 | C | CT | rs9526201 | 0.064 | -0.641[/]-0.704 | -0.639[/]-0.619 | -0.845[/]-0.858 | -1.413[/]-1.476 | -0.711[/]-0.714 |
| 13 | rs375520991 | 47310253 | C | CTGTGTGTG | rs9526201 | 0.06 | -1.489[/]-1.481 | -0.973[/]-0.989 | -1.093[/]-1.125 | -0.877[/]-0.822 | -0.873[/]-0.813 |
| 13 | rs842396 | 47274954 | G | C | rs9526201 | 0.059 | -0.844[/]-0.785 | -1.96[/]-1.96 | -1.645[/]-1.613 | -1.692[/]-1.688 | -1.667[/]-1.681 |
| 13 | rs35503529 | 47187521 | GA | G | rs9526201 | 0.058 | -1.709[/]-1.742 | -1.421[/]-1.479 | -1.248[/]-1.304 | -0.971[/]-0.996 | -0.855[/]-0.884 |
| 13 | rs10718141 | 47221929 | CT | C | rs9526201 | 0.054 | -0.314[/]-0.277 | 0.38[/]0.426 | 0.603[/]0.656 | 0.064[/]0.102 | 0.324[/]0.371 |
| 13 | rs79154774 | 47193247 | CT | C | rs9526201 | 0.053 | -2.164[/]-2.173 | -2.498[/]-2.501 | -1.961[/]-1.979 | -1.835[/]-1.886 | -1.735[/]-1.788 |
| 13 | rs138919787 | 47153598 | C | CT | rs9526201 | 0.042 | -1.392[/]-1.384 | -0.792[/]-0.762 | -0.683[/]-0.653 | -1.06[/]-1.038 | -1.484[/]-1.441 |
| 13 | rs5803355 | 47170002 | G | GTT | rs9526201 | 0.041 | -0.272[/]-0.313 | 0.179[/]0.174 | 0.213[/]0.206 | -0.047[/]-0.075 | 0.224[/]0.196 |
| 13 | rs371040869 | 47094955 | CT | C | rs9526201 | 0.038 | -1.014[/]-1.021 | -0.718[/]-0.738 | -0.868[/]-0.898 | -0.639[/]-0.646 | -1.291[/]-1.329 |
| 13 | rs34821427 | 47167418 | CT | C | rs9526201 | 0.026 | -1.049[/]-1.045 | -1.026[/]-1.053 | -0.686[/]-0.697 | -0.818[/]-0.826 | -0.737[/]-0.738 |
